# Supplementary material for: Direct observation of long-range chirality transfer in a self-assembled supramolecular monolayer at interface in situ
Source: Nat Commun. 2022 Dec 14;13:7737. doi: 10.1038/s41467-022-35548-z (PMC9750980; doi:10.1038/s41467-022-35548-z)
Supplement: Supplementary file 1 — Supplementary Information [file 41467_2022_35548_MOESM1_ESM.pdf]

Supplementary Information

Direct Observation of Long-Range Chirality Transfer in a Self-Assembled Supramolecular Monolayer at Interface in Situ

Yuening Zhang[1,2], Xujin Qin[1,2], Xuefeng Zhu[3], Minghua Liu[1,3], Yuan Guo[1,2], Zhen Zhang[1,2*]

[1] Beijing National Laboratory for Molecular Sciences, CAS Research/Education Center for Excellence in Molecular Sciences, Institute of Chemistry, Chinese Academy of Sciences, Beijing 100190, China.

[2] University of Chinese Academy Sciences, Beijing 100049, China.

[3] Beijing National Laboratory of Molecular Sciences, CAS Key Laboratory of Colloid, Interface and Thermodynamics, Institute of Chemistry, Chinese Academy of Sciences, Beijing 100190, China.

Contents

1. Principle and method of chiral SFG experiment S2
2. Air/water interfacial assembly of L-/D-GAn........S11
3. Brewster angle microscopy images of D-GAn monolayers........S13
4. Distinguish the peaks of three amide groups of glutamate........S14
5. L-GAn molecules form an antiparallel β-sheet-like structure at the air-water interface........S17
6. SFG spectra of L-/D-GAn monolayers in different surface pressures........S20
7. Orientation determination of antiparallel β-sheet-like........S22
8. Chiral SFG spectra in the region corresponding to C-H stretching vibrations...S24
9. Orientation determination of methyl groups........S27
10. Molecular snapshots of self-assembly........S30
11. Number of molecules assembled into nanorods........S32
12. Global fitting of SFG spectra in amide region........S35
13. Global fitting of SFG spectra in C-H region........S42
14. Synthesis of L-/D-GAn........S45
15. Supplementary References........S46

1. Principle and method of chiral SFG experiment

1.1 Vibrational sum-frequency generation

Sum-frequency vibration (SFG) spectroscopy is a second-order nonlinear optical technique, which principle has been widely reported in the literature.[1-5] SFG spectroscopy has two pulsed laser sources, infrared (IR) and visible (Vis) beams, that can generate SFG signals when the two beams achieve spatial and temporal overlap at the interface. For a certain polarization setting, the SFG intensity I_{SFG} can be described by effective second-order susceptibility,

$$I_{SFG} \propto \left|\chi_{eff}^{(2)}\right|^2 I_{Vis} I_{IR} \tag{1}$$

The second-order susceptibility ($\chi_{eff}^{(2)}$) of an interface consists of a non-resonant term and resonant terms, as follows,

$$\chi_{eff}^{(2)} = \chi_{NR}^{(2)} + \sum_q \chi_q^{(2)} = \chi_{NR}^{(2)} + \sum_q \frac{A_q}{\omega_{IR} - \omega_q + i\Gamma_q} \tag{2}$$

in which $\chi_{NR}^{(2)}$ is the nonresonant susceptibility, which is typically very small in the air/water interface experiments. A_q, ω_q, and Γ_q are the amplitude, resonant frequency, and Lorentzian width of the qth vibrational mode respectively. To reveal the vibrational signatures of the interfacial molecules, SFG spectra are commonly plotted as a function of the incident IR wavenumber (in cm^{-1}). In this paper, we used eq. 2 to perform global fitting of spectral peaks.

As shown in Supplementary Figs. 1a-b, we performed sss polarization measurement of the D-GAn monolayer in the amide and C-H regions in the rotation sample cell. The results showed that the sss signal was zero (χ_{XXX} is zero), which confirmed that the L-/D-GAn monolayer was an isotropic interface with C_∞ symmetry[6,7]. In addition, we also measured the ssp and sss polarization spectra of the D-GAn monolayer at high surface pressure (Supplementary Fig. 1c) to further check the anisotropy of the interface. The results confirmed that no sss signal was detected neither due to experimental error nor low signal-to-noise ratio. Therefore, the L-/D-GAn self-assemblies are isotropic at the interface and the self-assembly did not form the microcrystal.

It must be pointed out that we used a broadband SFG (resolution ~8 cm^{-1}) to do the

additional sss polarization measurement to avoid the high laser intensity damage to the samples in the high-resolution sum-frequency generation (HR-SFG, resolution 0.4 cm^{-1}) we used.

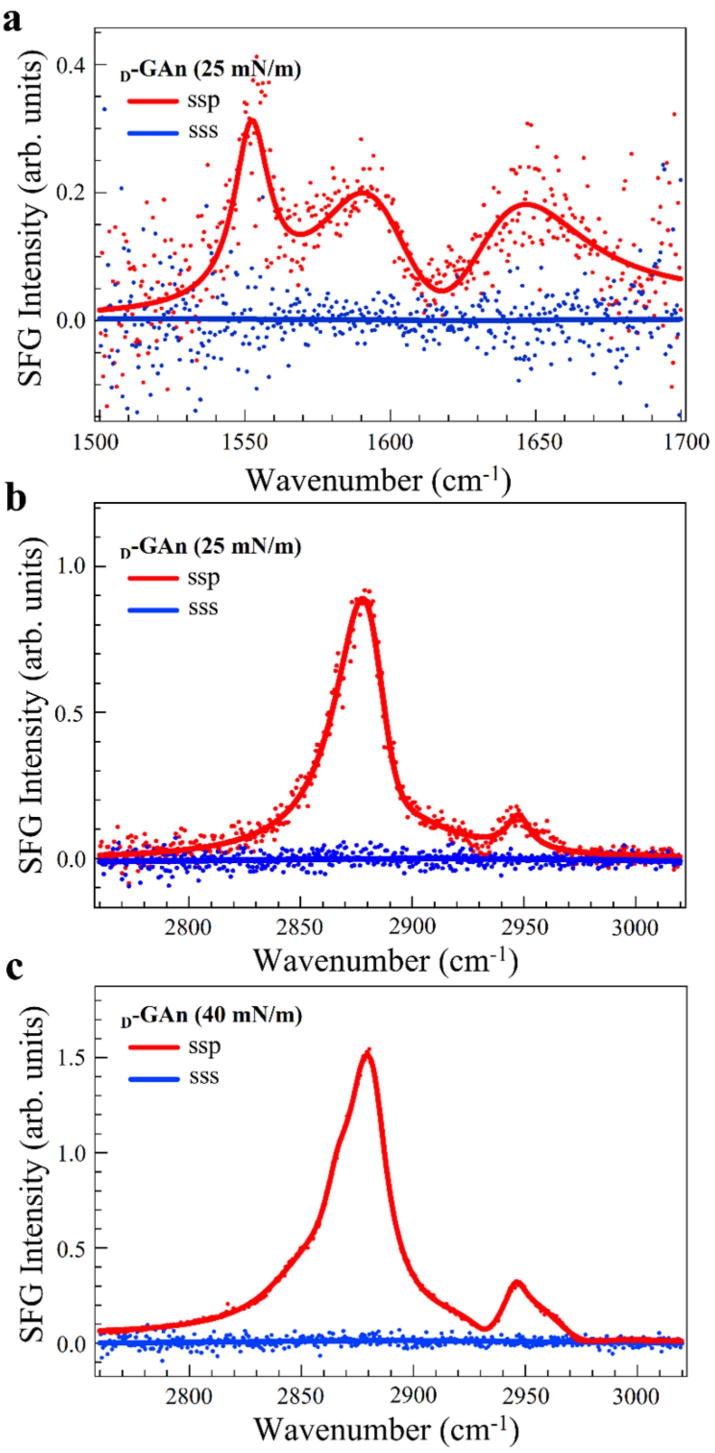

Supplementary Fig. 1 SFG spectral characteristics of D-GAn assemblies in the amide and C-H regions. Broadband sum-frequency generation vibrational spectra were recorded using sss and ssp polarization combinations for D-GAn monolayer in (**a**) amide region under 25 mN/m and C-H

region under (**b**) 25 mN/m and (**c**) 40 mN/m at the air/water interface.

1.2 SFG-VS polarization combinations

In vibrational SFG, the sample interacts with a tunable IR beam with frequency ω_{IR} and polarization $\hat{K}$, and with a non-resonant visible beam with frequency ω_{Vis} and polarization $\hat{J}$. The SFG signal is emitted with frequency $\omega_{SFG} = \omega_{Vis} + \omega_{IR}$ and polarization $\hat{I}$. Typically, we define the Z-axis as the surface normal, and the X-Z plane as the scattering plane. The beams are polarized either in the X-Z plane (p-polarization) or in the Y-direction (s-polarization). The angles of the input and sum-frequency wavevectors with respect to the surface normal are β_{IR}, β_{Vis}, and β_{SFG} (the β is between 0 and 90° for all three beams). All these definitions apply to the laboratory frame, symbolized here using capital letters (X/Y/Z and I/J/K).

We focused our derivation on the lab-frame ssp (s-polarized SFG, s-polarized visible beam, and p-polarized IR beam), sps, ppp and psp, spp, pps signals, which consist of three types of factors: elements of the (second-rank) Fresnel tensor L, which describe effects caused by the different refractive indices of the media under study; factors arising from the angles of the input and sum-frequency beams; and elements of the complex third-rank tensor χ (individual elements are symbolized as $\chi_{IJK}^{(2)}$), which describe the intrinsic response of the sample. We symbolize the full lab-frame response (including all three effects) as $\chi_{eff}^{(2)}$, the "effective" response. $\chi_{eff}^{(2)}$ is related to χ by the equation S3:

$$\chi_{eff}^{(2)} = \left[\hat{I} \cdot L(\omega_{SFG}, \beta_{SFG})\right] \cdot \chi : \left\{\left[\hat{J} \cdot L(\omega_{Vis}, \beta_{Vis})\right]\left[\hat{K} \cdot L(\omega_{IR}, \beta_{IR})\right]\right\} \quad (3)$$

To write the final expressions for the effective ssp, sps, ppp and psp, spp, pps signals, $\chi_{eff,ssp}^{(2)}$, $\chi_{eff,sps}^{(2)}$, $\chi_{eff,ppp}^{(2)}$ and $\chi_{eff,psp}^{(2)}$, $\chi_{eff,spp}^{(2)}$, $\chi_{eff,pps}^{(2)}$ note that s-polarization corresponds to $\hat{Y}$- polarization in the lab frame, whereas p-polarization corresponds to polarization in the direction $cos\beta_{IR}\hat{X} + sin\beta_{IR}\hat{Z}$ for the IR beam, $cos\beta_{Vis}\hat{X} + sin\beta_{Vis}\hat{Z}$ for the visible beam, or $-cos\beta_{SFG}\hat{X} + sin\beta_{SFG}\hat{Z}$ for the sum-frequency beam. Substituting these polarization vectors into eq. 3, the nonzero $\chi_{IJK}^{(2)}$ elements of an interface can be measured by manipulating the polarization of incident beams and

SFG signals:

$$\chi^{(2)}_{eff,ssp}$$

$$= L_{YY}(\omega_{SFG},\beta_{SFG})L_{YY}(\omega_{Vis},\beta_{Vis})L_{XX}(\omega_{IR},\beta_{IR})\ cos\beta_{IR}\ \chi^{(2)}_{YYX}$$

$$+L_{YY}(\omega_{SFG},\beta_{SFG})L_{YY}(\omega_{Vis},\beta_{Vis})L_{ZZ}(\omega_{IR},\beta_{IR})sin\beta_{IR}\chi^{(2)}_{YYZ} \quad (4)$$

$$\chi^{(2)}_{eff,sps}$$

$$= L_{YY}(\omega_{SFG},\beta_{SFG})L_{ZZ}(\omega_{Vis},\beta_{Vis})L_{YY}(\omega_{IR},\beta_{IR})sin\beta_{Vis}\chi^{(2)}_{YZY}$$

$$+L_{YY}(\omega_{SFG},\beta_{SFG})L_{XX}(\omega_{Vis},\beta_{Vis})L_{YY}(\omega_{IR},\beta_{IR})cos\beta_{Vis}\chi^{(2)}_{YXY} \quad (5)$$

$$\chi^{(2)}_{eff,ppp}$$

$$= -L_{XX}(\omega_{SFG},\beta_{SFG})L_{XX}(\omega_{Vis},\beta_{Vis})L_{XX}(\omega_{IR},\beta_{IR})cos\beta_{SFG}cos\beta_{Vis}\ cos\beta_{IR}\ \chi^{(2)}_{XXX}$$

$$-L_{XX}(\omega_{SFG},\beta_{SFG})L_{XX}(\omega_{Vis},\beta_{Vis})L_{ZZ}(\omega_{IR},\beta_{IR})cos\beta_{SFG}cos\beta_{Vis}\ sin\beta_{IR}\ \chi^{(2)}_{XXZ}$$

$$-L_{XX}(\omega_{SFG},\beta_{SFG})L_{ZZ}(\omega_{Vis},\beta_{Vis})L_{XX}(\omega_{IR},\beta_{IR})cos\beta_{SFG}sin\beta_{Vis}cos\beta_{IR}\chi^{(2)}_{XZX}$$

$$-L_{XX}(\omega_{SFG},\beta_{SFG})L_{ZZ}(\omega_{Vis},\beta_{Vis})L_{ZZ}(\omega_{IR},\beta_{IR})cos\beta_{SFG}sin\beta_{Vis}sin\beta_{IR}\chi^{(2)}_{XZZ}$$

$$+L_{ZZ}(\omega_{SFG},\beta_{SFG})L_{XX}(\omega_{Vis},\beta_{Vis})L_{XX}(\omega_{IR},\beta_{IR})sin\beta_{SFG}cos\beta_{Vis}cos\beta_{IR}\chi^{(2)}_{ZXX}$$

$$+L_{ZZ}(\omega_{SFG},\beta_{SFG})L_{XX}(\omega_{Vis},\beta_{Vis})L_{ZZ}(\omega_{IR},\beta_{IR})sin\beta_{SFG}cos\beta_{Vis}sin\beta_{IR}\chi^{(2)}_{ZXZ}$$

$$+L_{ZZ}(\omega_{SFG},\beta_{SFG})L_{ZZ}(\omega_{Vis},\beta_{Vis})L_{XX}(\omega_{IR},\beta_{IR})sin\beta_{SFG}sin\beta_{Vis}cos\beta_{IR}\chi^{(2)}_{ZZX}$$

$$+L_{ZZ}(\omega_{SFG},\beta_{SFG})L_{ZZ}(\omega_{Vis},\beta_{Vis})L_{ZZ}(\omega_{IR},\beta_{IR})sin\beta_{SFG}sin\beta_{Vis}sin\beta_{IR}\chi^{(2)}_{ZZZ} \quad (6)$$

$$\chi^{(2)}_{eff,psp}$$

$$= L_{ZZ}(\omega_{SFG},\beta_{SFG})L_{YY}(\omega_{Vis},\beta_{Vis})L_{ZZ}(\omega_{IR},\beta_{IR})sin\beta_{SFG}sin\beta_{IR}\chi^{(2)}_{ZYZ}$$

$$+L_{ZZ}(\omega_{SFG},\beta_{SFG})L_{YY}(\omega_{Vis},\beta_{Vis})L_{XX}(\omega_{IR},\beta_{IR})sin\beta_{SFG}cos\beta_{IR}\chi^{(2)}_{ZYX}$$

$$-L_{XX}(\omega_{SFG},\beta_{SFG})L_{YY}(\omega_{Vis},\beta_{Vis})L_{ZZ}(\omega_{IR},\beta_{IR})cos\beta_{SFG}sin\beta_{IR}\chi^{(2)}_{XYZ}$$

$$-L_{XX}(\omega_{SFG},\beta_{SFG})L_{YY}(\omega_{Vis},\beta_{Vis})L_{XX}(\omega_{IR},\beta_{IR})cos\beta_{SFG}cos\beta_{IR}\chi^{(2)}_{XYX} \quad (7)$$

$$\chi^{(2)}_{eff,spp}$$

$$= L_{YY}(\omega_{SFG}, \beta_{SFG}) L_{ZZ}(\omega_{Vis}, \beta_{Vis}) L_{XX}(\omega_{IR}, \beta_{IR}) sin\beta_{Vis} cos\beta_{IR} \chi_{YZX}^{(2)}$$

$$+L_{YY}(\omega_{SFG}, \beta_{SFG}) L_{XX}(\omega_{Vis}, \beta_{Vis}) L_{XX}(\omega_{IR}, \beta_{IR}) cos\beta_{Vis} cos\beta_{IR} \chi_{YXX}^{(2)}$$

$$+L_{YY}(\omega_{SFG}, \beta_{SFG}) L_{XX}(\omega_{Vis}, \beta_{Vis}) L_{ZZ}(\omega_{IR}, \beta_{IR}) cos\beta_{Vis} sin\beta_{IR} \chi_{YXZ}^{(2)}$$

$$+L_{YY}(\omega_{SFG}, \beta_{SFG}) L_{ZZ}(\omega_{Vis}, \beta_{Vis}) L_{ZZ}(\omega_{IR}, \beta_{IR}) sin\beta_{Vis} sin\beta_{IR} \chi_{YZZ}^{(2)} \quad (8)$$

$\chi_{eff,pps}^{(2)}$

$$= -L_{XX}(\omega_{SFG}, \beta_{SFG}) L_{XX}(\omega_{Vis}, \beta_{Vis}) L_{YY}(\omega_{IR}, \beta_{IR}) cos\beta_{SFG} cos\beta_{Vis} \chi_{XXY}^{(2)}$$

$$-L_{XX}(\omega_{SFG}, \beta_{SFG}) L_{ZZ}(\omega_{Vis}, \beta_{Vis}) L_{YY}(\omega_{IR}, \beta_{IR}) cos\beta_{SFG} sin\beta_{Vis} \chi_{XZY}^{(2)}$$

$$+L_{ZZ}(\omega_{SFG}, \beta_{SFG}) L_{XX}(\omega_{Vis}, \beta_{Vis}) L_{YY}(\omega_{IR}, \beta_{IR}) sin\beta_{SFG} cos\beta_{Vis} \chi_{ZXY}^{(2)}$$

$$+L_{ZZ}(\omega_{SFG}, \beta_{SFG}) L_{ZZ}(\omega_{Vis}, \beta_{Vis}) L_{YY}(\omega_{IR}, \beta_{IR}) sin\beta_{SFG} sin\beta_{Vis} \chi_{ZZY}^{(2)} \quad (9)$$

For an chiral surface with C_∞ symmetry, there are thirteen nonzero $\chi_{IJK}^{(2)}$ elements, of which seven achiral elements ($\chi_{XXZ}^{(2)} = \chi_{YYZ}^{(2)}$, $\chi_{XZX}^{(2)} = \chi_{YZY}^{(2)}$, $\chi_{ZXX}^{(2)} = \chi_{ZYY}^{(2)}$, $\chi_{ZZZ}^{(2)}$) and six chiral elements ($\chi_{XYZ}^{(2)}$, $\chi_{YXZ}^{(2)}$, $\chi_{ZXY}^{(2)}$, $\chi_{ZYX}^{(2)}$, $\chi_{XZY}^{(2)}$, $\chi_{YZX}^{(2)}$), so effective second-order susceptibility tensor elements, $\chi_{eff,ssp}^{(2)}$, $\chi_{eff,sps}^{(2)}$, $\chi_{eff,ppp}^{(2)}$ and $\chi_{eff,psp}^{(2)}$, $\chi_{eff,spp}^{(2)}$, $\chi_{eff,pps}^{(2)}$ in eq. 4-9 can be simplified:

$\chi_{eff,ssp}^{(2)}$

$$= L_{YY}(\omega_{SFG}) L_{YY}(\omega_{VIS}) L_{ZZ}(\omega_{IR}) sin\beta_{IR} \chi_{YYZ}^{(2)} \quad (10)$$

$\chi_{eff,sps}^{(2)}$

$$= L_{YY}(\omega_{SFG}) L_{ZZ}(\omega_{VIS}) L_{YY}(\omega_{IR}) sin\beta_{VIS} \chi_{YZY}^{(2)} \quad (11)$$

$\chi_{eff,ppp}^{(2)}$

$$= -L_{XX}(\omega_{SFG}) L_{XX}(\omega_{VIS}) L_{ZZ}(\omega_{IR}) cos\beta_{SFG} cos\beta_{VIS} sin\beta_{IR} \chi_{XXZ}^{(2)}$$

$$-L_{XX}(\omega_{SFG}) L_{ZZ}(\omega_{VIS}) L_{XX}(\omega_{IR}) cos\beta_{SFG} sin\beta_{VIS} cos\beta_{IR} \chi_{XZX}^{(2)}$$

$$+L_{ZZ}(\omega_{SFG}) L_{XX}(\omega_{VIS}) L_{XX}(\omega_{IR}) sin\beta_{SFG} cos\beta_{VIS} cos\beta_{IR} \chi_{ZXX}^{(2)}$$

$$+L_{ZZ}(\omega_{SFG})L_{ZZ}(\omega_{VIS})L_{ZZ}(\omega_{IR})sin\beta_{SFG}sin\beta_{VIS}sin\beta_{IR}\chi^{(2)}_{ZZZ} \quad (12)$$

$$\chi^{(2)}_{eff,psp}$$
$$= L_{ZZ}(\omega_{SFG})L_{YY}(\omega_{VIS})L_{XX}(\omega_{IR})sin\beta_{SFG}cos\beta_{IR}\chi^{(2)}_{ZYX}$$
$$-L_{XX}(\omega_{SFG})L_{YY}(\omega_{VIS})L_{ZZ}(\omega_{IR})cos\beta_{SFG}sin\beta_{IR}\chi^{(2)}_{XYZ} \quad (13)$$

$$\chi^{(2)}_{eff,spp}$$
$$= L_{YY}(\omega_{SFG})L_{ZZ}(\omega_{VIS})L_{XX}(\omega_{IR})sin\beta_{VIS}cos\beta_{IR}\chi^{(2)}_{YZX}$$
$$-L_{YY}(\omega_{SFG})L_{XX}(\omega_{VIS})L_{ZZ}(\omega_{IR})cos\beta_{SFG}sin\beta_{VIS}\chi^{(2)}_{YXZ} \quad (14)$$

$$\chi^{(2)}_{eff,pps}$$
$$= L_{ZZ}(\omega_{SFG})L_{XX}(\omega_{VIS})L_{YY}(\omega_{IR})sin\beta_{SFG}cos\beta_{VIS}\chi^{(2)}_{ZXY}$$
$$-L_{XX}(\omega_{SFG})L_{ZZ}(\omega_{VIS})L_{YY}(\omega_{IR})cos\beta_{SFG}sin\beta_{VIS}\chi^{(2)}_{XZY} \quad (15)$$

1.3 Molecular hyperpolarizability and calculation of group orientation

In order to calculate the molecular orientation information, we introduced the molecular hyperpolarizability (*β*), which determines the SFG response of a molecule. The $\chi^{(2)}_{IJK}$ tensor elements can be expressed in terms of the $\beta^{(2)}_{ijk}$ tensor elements using the Euler transformation:

$$\chi^{(2)}_{IJK,q} = N\sum_{i,j,k}\langle R_{Ii}R_{Jj}R_{Kk}\rangle\beta^{(2)}_{ijk,q} \quad (16)$$

where I, J, K are the laboratory coordinates (X, Y, Z) and i, j, k are molecular coordinates (a, b, c); *N* is the number density of the molecular moiety under study; R_{Ii}, R_{Jj} and R_{Kk} are elements of the rotational transformation matrix connecting the molecular coordinates to the laboratory coordinates. In this work, the effective second-order susceptibility correlates to the hyperpolarizability (*β*) tensor of molecules at interfaces by using the z-y-z Euler transformation. The z-y-z transformation is achieved by the clockwise rotation of the molecular c axis by ψ, b axis by θ, and c axis by ϕ to overlap with the laboratory coordinates. For a monolayer with a random orientation distribution within the surface plane, the Euler transformation introduces the molecular

orientation (θ, ψ), while the in-plane rotation angle (ϕ) is averaged by the integration over 0 to 2π for the isotropic interface, with $sin^2\phi = cos^2\phi = \frac{1}{2}$ and $cos\,\phi\, sin\,\phi = 0$.[8]

1.4 Chiral SFG-VS spectroscopy of amide modes

The hydrogen bond formed between different amide groups usually gives four different bands, i.e. amide I (primarily CO stretch), amide II (CN stretch and NH in-plane bend), amide III (CN stretch, NH bend, and CO in-plane bend), and amide A (NH stretch).[9] Previous studies have shown the amide I band is sensitive to the structures and amount of secondary structures and the local environments which are not strongly influenced by side chains.[10] The α-helical, parallel, and antiparallel β-sheet structures are the most widely encountered secondary structures in peptides and proteins, and different types of secondary structures show different peak centers. In order to deduce the type and orientation of secondary structure from SFG signals obtained using various polarization combinations, we need to know the second-order surface susceptibility of interfacial peptides in the lab coordinate system and the hyperpolarizability in the molecular coordinate system associated with it. In this work, we used two chiral polarization combinations of psp and spp to detect chiral amide I stretch. The intensity of the chiral SFG signal measured by the psp polarization is related to $\chi^{(2)}_{eff,psp}$ and consequently $\chi^{(2)}_{ZYX}$, and $\chi^{(2)}_{XYZ}$ in the chiral interface with C_∞ symmetry, as shown in eq. 13. Similarly, the intensity of the chiral SFG signal measured using the spp polarization is related to $\chi^{(2)}_{eff,spp}$ and consequently $\chi^{(2)}_{YZX}$, and $\chi^{(2)}_{YXZ}$, eq. 14.

α-helical structure and $C_{\frac{18}{5}}$ symmetry

The work of the Chen group has shown that both the A mode and E_1 mode of α-helix amide I stretching can contribute to SFG signals, and the repeating unit of α-helix follow $C_{\frac{18}{5}}$ symmetry.[11,12] The nonzero hyperpolarizability tensor elements for the A mode are β_{ccc}, $\beta_{aac} = \beta_{bbc}$. We can deduce the susceptibility tensor elements for the A mode of α-helices:

$$\chi_{A,ZYX} = \chi_{A,YZX} = \chi_{A,XYZ} = \chi_{A,YXZ} = 0 \qquad (17)$$

The nonzero hyperpolarizability tensor elements for the E_1 mode of a-helices are $\beta_{caa} = \beta_{cbb} = \beta_{aca} = \beta_{bcb}$, $\beta_{acb} = -\beta_{bca} = \beta_{cab} = -\beta_{cba}$, and the susceptibility tensor elements for the E_1 mode:

$$\chi_{E_1,ZYX} = \chi_{E_1,YZX} = \frac{1}{2}N_s(1 - 3cos^2\theta)\beta_{acb} \qquad (18)$$

$$\chi_{E_1,XYZ} = \chi_{E_1,YXZ} = 0 \qquad (19)$$

Where N_S is the surface density of a-helical repeat units. From the above derivation, it can be seen that $\chi^{(2)}_{eff,psp} = \chi^{(2)}_{eff,spp} = 0$ for the A mode and $\chi^{(2)}_{eff,psp} = \chi^{(2)}_{eff,spp} = \frac{1}{2}N_s(1 - 3cos^2\theta)\beta_{acb}$ for E_1 mode of the a-helical structure.

Parallel β-sheet structure and C_2 symmetry

A parallel β-sheet is characterized by two peptide strands running in the same direction held together by hydrogen bonding between the strands. The repeat unit has C_2 symmetry, thus two modes of amides I are expected: A and B modes.[13] Since the signal derived from the contribution of the B mode of the amide I band of parallel β-sheet usually appears at a lower frequency (about 1620 cm^{-1}), it does not match the signal peak position of the amide I band we observed in our experiment, so here we only discussed the A mode of parallel β-sheet structure.

The nonzero hyperpolarizability tensor elements for the A mode of parallel β-sheet are β_{aab}, β_{ccb}, β_{bbb}, $\beta_{acb} = \beta_{cab}$, and the susceptibility tensor elements for the A mode:

$$\chi_{A,ZYX} = \chi_{A,YZX} = \frac{1}{2}N_s[sin\,\theta\,cos\,\theta\,cos\,\psi\,(\beta_{aab} - \beta_{ccb}) + (sin^2\theta cos^2\psi - cos^2\theta)\beta_{cab}] \qquad (20)$$

$$\chi_{A,XYZ} = \chi_{A,YXZ} = 0 \qquad (21)$$

Where N_S is the surface density of parallel β-sheet repeat units. From the above derivation, it can be seen that $\chi^{(2)}_{eff,psp} = \chi^{(2)}_{eff,spp}$ for the A mode of the parallel β-sheet structure.

Antiparallel β-sheet structure and D_2 symmetry

The antiparallel β-sheet structure follows D_2 symmetry, in which the susceptibility tensor elements of different modes and orientation analysis are discussed in detail in Supplementary Section 7 (Orientation determination of antiparallel β-sheet). For B_2 mode of antiparallel β-sheet structure, $\chi^{(2)}_{eff,psp} = \chi^{(2)}_{eff,spp} = -\frac{1}{2}N_s(cos^2\theta - sin^2\theta cos^2\psi)\beta_{acb}$.

1.5 Interference chiral polarization combinations

In this work, we monitored the SFG spectra in the C-H and amide region of L-GAn and D-GAn monolayers in different surface pressure at the air/water interface using the ssp, sps, ppp achiral polarization combinations and psp, spp, s(+m)p-s(-m)p, p(+m)p-p(-m)p chiral polarization combinations to investigate the supramolecular chiral information transfer during fabricating the self-assembled supramolecular monolayer. We can use the psp polarization combination to measure the interface chirality in the amide region, but in the C-H region, the chiral signal is weak, and it is difficult to use the chiral polarization combination of psp, spp, and pps to detect. We measured the weaker chiral SFG spectra in the C-H region by using the interference method of s(+m)p-s(-m)p polarization. Where the m (usually 45°) denotes the mixed s and p polarization, + denotes clockwise rotation from the p polarization in the incident plane facing the incoming beam, and - denotes counterclockwise rotation from the p polarization. By exploiting the s(+m)p-s(-m)p polarization, the achiral SFG field will be introduced to interfere with the chiral SFG signals, so that the chiral part could be enhanced in comparison to the direct measurement of the much smaller $\left|\chi^{(2)}_{spp}\right|^2$ term. The interference crossing term for s(+m)p-s(-m)p is,

$$I_{s(+m)p} - I_{s(-m)p} \propto Re\left\{\chi^{(2)}_{ssp}\chi^{(2)}_{spp}\,^*\right\} \quad (22)$$

Similarly, the interference crossing term for p(+m)p-p(-m)p is,

$$I_{p(+m)p} - I_{p(-m)p} \propto Re\left\{\chi^{(2)}_{ppp}\chi^{(2)}_{psp}\,^*\right\} \quad (23)$$

As stated in the equation above, the s(+m)p-s(-m)p spectra measure the actual value

of the product of achiral ssp and chiral spp magnetic susceptibility terms, and the p(+m)p-p(-m)p spectra measure the actual value of the product of achiral ppp and chiral psp magnetic susceptibility terms. And because L-GAn, D-GAn, and racemic mixtures (50/50 equivalent mixtures) show the same ssp and ppp SFG spectra at the air/water interface, that is, L-GAn and D-GAn actually have the same effective achiral susceptibility.[14] Therefore, the p(+m)p-p(-m)p spectrum is a direct measurement of the relationship between the intensity and the sign of the chiral psp term.

2. Air/water interfacial assembly of L-/D-GAn

The phase behavior of L-GAn at the air/water interface was investigated by surface pressure measurements (isotherm experiments) and Brewster angle microscopy. The surface pressure (π) is defined as the interfacial tension difference between a clean water interface (γ_0) and an interface in the presence of the amphiphilic molecule (γ); the expression is $\pi = \gamma_0 - \gamma$. L-GAn molecules are spread on the water surface and compressed by the barriers. The surface pressure (π) is plotted as a function of the area (A) per molecule at a constant temperature. The π-A isotherms can reflect phase states, such as gas (G), liquid (L), and solid (S) and various mesophases between ideal liquid and solid states at the air-water interface during compression.

L-/D-GAn contains two hydrophobic alkyl chains, an anthracene unit, and a hydrophilic glutamic acid group as shown in Supplementary Fig. 2. There are three amide groups in the glutamic acid unit which are usually easily formed inter-molecular hydrogen-bond,[15] and the anthracene group can easily stack through strong π-π interaction, which assists the molecules to form the self-assembled nanostructures.[16] Supplementary Fig. 2 shows the compression isotherms (π-A) of the L-GAn and D-GAn spreading on the water subphase at 25°C. The phase transition properties of L-/D-GAn on the water at room temperature are divided into several stages, (i) the gas-liquid expanded coexistence region (not shown, G-LE, >64 Å^2), (ii) the liquid expanded (LE, 57-64 Å^2) phase, (iii) the liquid expanded-liquid condensed coexistence region (LE-LC, 38-57 Å^2), (iv) the liquid condensed (LC, 10-38 Å^2) phase, and (v) the collapse phase (not shown, <10 Å^2) are observed. Both L-GAn and D-GAn monolayers show the onset

of surface pressure at 64 Å^2 per molecule. As the L-/D-GAn monolayer is compressed to the LE phase, the surface pressure starts to rise. The LE-LC phase transition plateau occurs at about 5 to 6 mN/m. As the surface pressure continues to rise, the mean molecular area (MMA) gradually decreases, and the monolayer becomes denser, as shown in the Brewster angle microscope images on the right of Supplementary Fig. 2. Then, the surface pressure continues to increase with compression at LC phase, indicating a more rigid packing state of L-/D-GAn molecules.

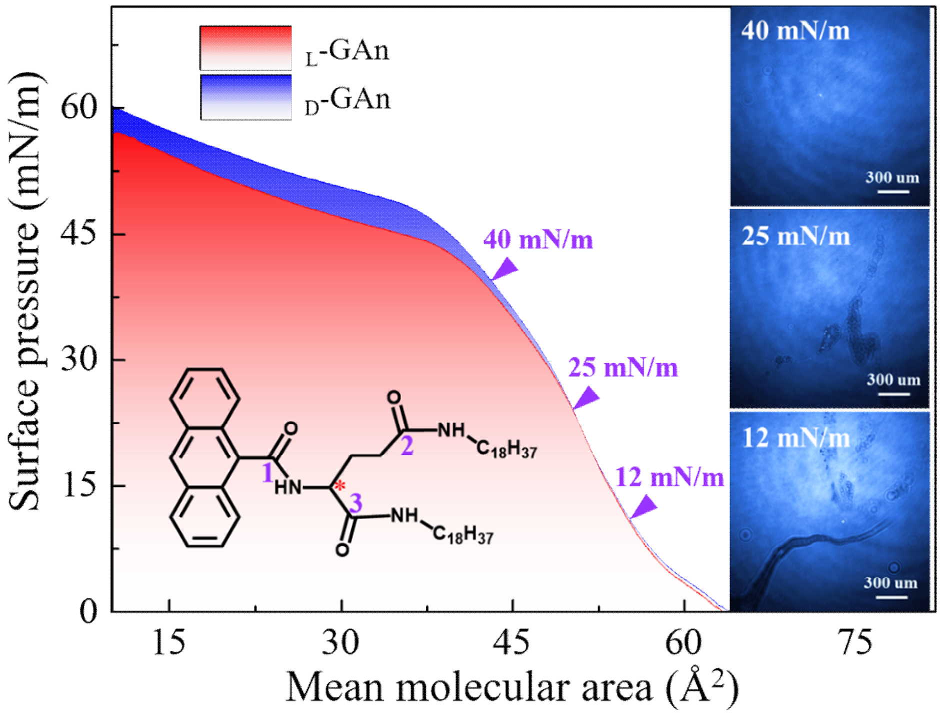

Supplementary Fig. 2 L-/D-GAn molecules formed supramolecular assemblies. Surface pressure-molecular area (π-A) isotherms of L-GAn and D-GAn on a pure water subphase at 25°C. The illustration on the left shows the L-/D-GAn molecular structure, the sequence numbers (1, 2, 3) represent three amide bonds in different positions of the molecule, and * represents the chiral center. The illustration on the right shows Brewster angle microscopy images of D-GAn monolayers in three surface pressures.

3. Brewster angle microscopy images of D-GAn monolayers

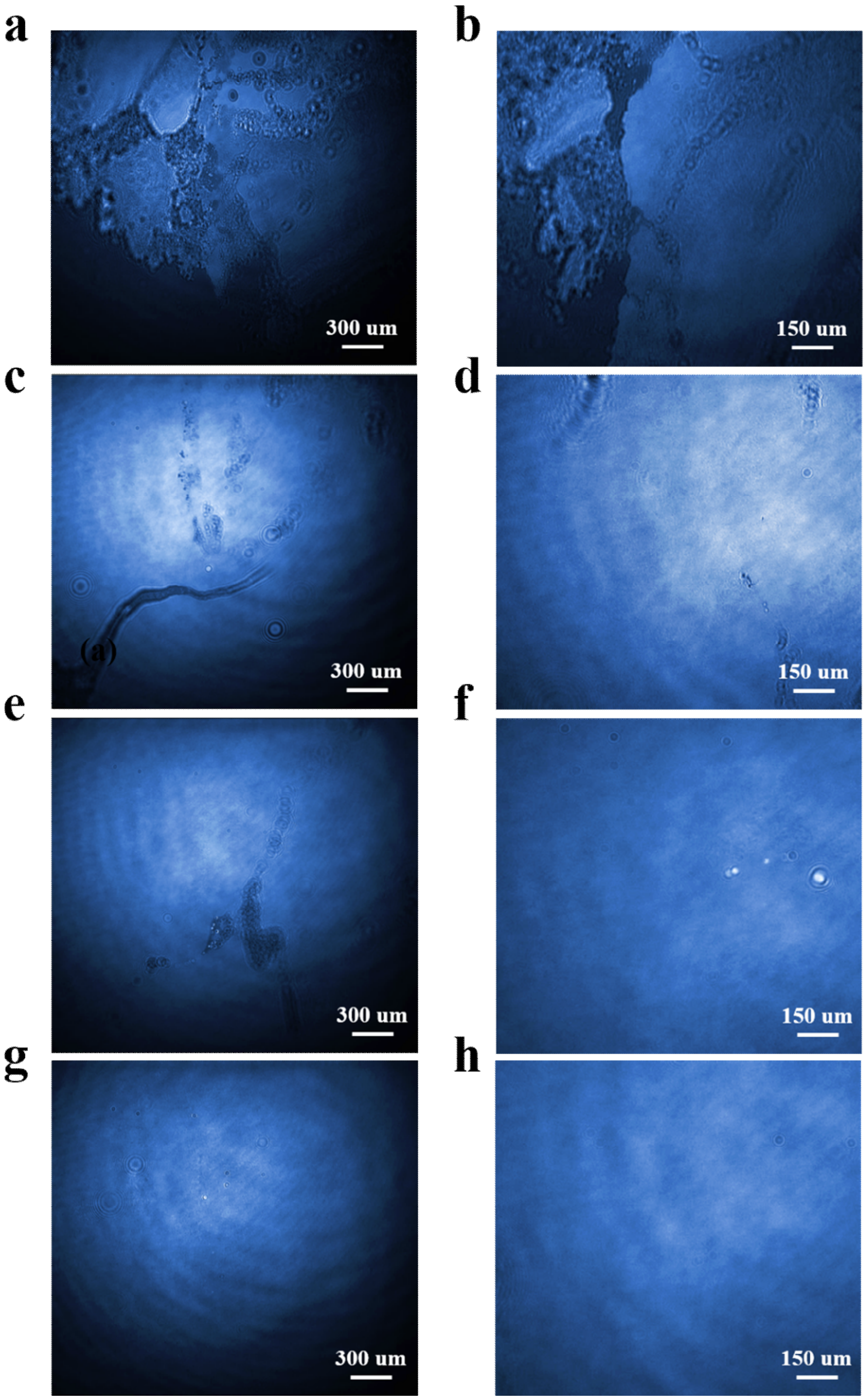

Supplementary Fig. 3 Brewster angle microscopy images of D-GAn monolayer in different surface pressures. a and (**b**) 4 mN/m, (**c**) and (**d**) 12 mN/m, (**e**) and (**f**) 25 mN/m, (**g**) and (**h**) 40 mN/m.

4. Distinguish the peaks of three amide groups of glutamate

The amide I vibrational mode arises mainly from the C=O stretching vibration, with minor contributions from the out-of-phase CN stretching vibration, CN deformation, and NH in-plane bending and is known to be intrinsically sensitive to the peptide or protein backbone and secondary structures at or above approximately 1600 cm^{-1}.[17] The amide II mode is the out-of-phase combination of the NH in-plane bending and CN stretching vibrations, with smaller contributions from the CO in-plane bending and the CC and NC stretching vibrations. However, the correlation between the secondary structure and the frequency of the amide II band is less straightforward than that for the amide I vibration[18].

As shown in Supplementary Fig. 4a, the molecular structure of the Fmoc-Glu-C18 and Fmoc-Asp-C17 is very similar, and the main difference is that the number of methylene groups that are connected between the amide 2 and the chiral carbon. Because the molecular structure of the two molecules is highly similar, we believe that the two molecules are spread as monolayers on the interface in a similar assembly structure. By comparing the signal peaks of the two monolayers in the amide band (Supplementary Figs. 4b-c), we deduced that the two peaks with significant shifts should be assigned to the amide I and amide II band of amide 2 respectively, that is, the strong signal peaks at 1555 and 1647 cm^{-1} can be assigned to the amide II and amide I band of amide 2 of Fmoc-Glu-C18, respectively.

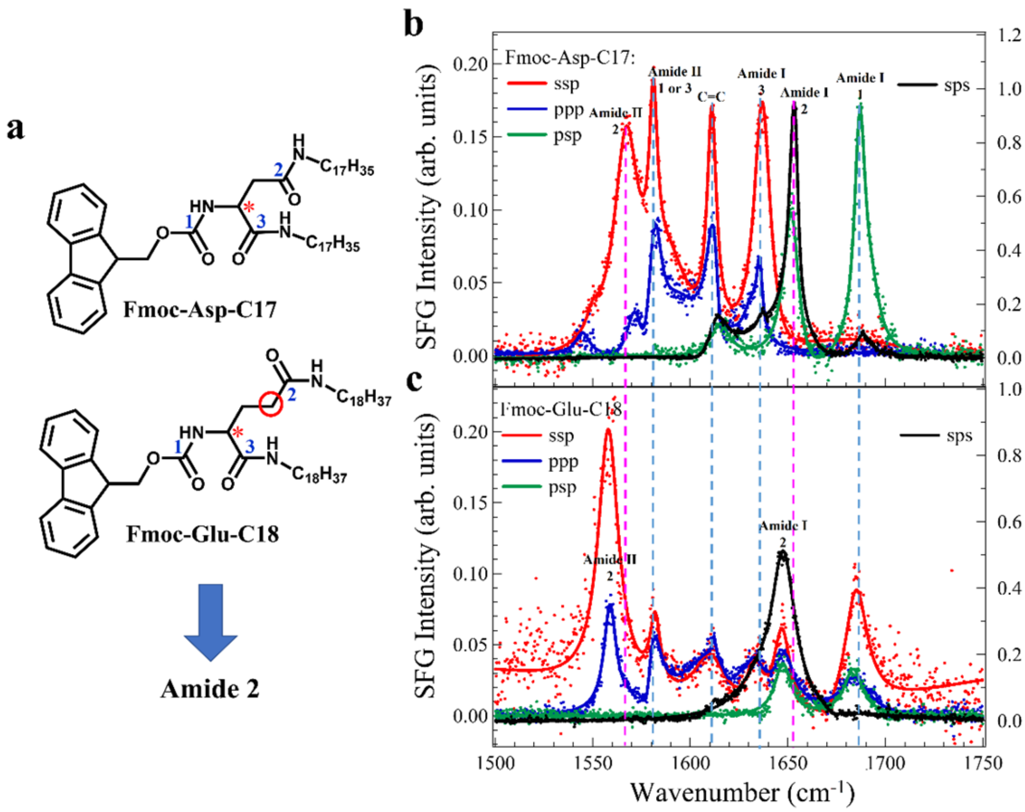

Supplementary Fig. 4 Assign the signal peak of amide 2. a, The molecular structures of Fmoc-Asp-C17 and Fmoc-Glu-C18. SFG-VS spectra were recorded using achiral ssp, sps, ppp polarization combinations, and chiral psp polarization combination for (**b**) Fmoc-Asp-C17 and (**c**) Fmoc-Glu-C18 monolayers (40 mN/m) at the air/water interface. (In Supplementary Figs. 4b-c, the spectra measured under the ssp, ppp, psp polarization combinations used the left-hand coordinate system, and the spectrum measured under the sps polarization combination used the right-hand coordinate system.)

Using a similar analysis method as above, we selected two molecules of D-GAn and Fmoc-Glu-C18 as a control group to distinguish the amide I and amide II band of amide 1. As shown in Supplementary Fig. 5a, for the molecular structure of Fmoc-Glu-C18, the steric hindrance between the aromatic ring and amide 1 was diminished by the spacer than D-GAn. In addition, the carbon-carbon stretching vibration of the fluorenylmethoxycarbonyl protecting group (Fmoc group) and anthracene group is located at 1610 and 1620 cm^{-1} respectively, through the infrared spectra search in the National Institute of Standards and Technology (NIST). By comparing the signal peaks of the two monolayers in the amide band (Supplementary Figs. 5b-c), we concluded that the three peaks with significant shifts should be assigned to the amide I, amide II

band of amide 1, and carbon-carbon stretching vibration of aromatic ring respectively, that is, the signal peaks at 1598 and 1657 cm^{-1} can be assigned to the amide II and amide I band of amide 1 of D-GAn, respectively.

In summary, by comparing the peaks of D-GAn and Fmoc-Glu-C18 in the amide band (Figs. 1d and f, and Supplementary Figs. 5b and c), the peaks at 1657 and 1598 cm^{-1} are assigned to the amide I and II bands of amide 1 of D-GAn. In the same way, by comparing the spectra of the Fmoc-Asp-C17 and Fmoc-Glu-C18 monolayers (Supplementary Figs. 4b and c), the peaks at 1653 and 1568 cm^{-1} are assigned to the amide I and II bands of amide 2 of Fmoc-Asp-C17, and the peaks at 1647 and 1555 cm^{-1} are assigned to the amide I and II bands of amide 2 of Fmoc-Glu-C18, respectively. The other two amide peaks of D-GAn at 1637 and 1578 cm^{-1} are assigned to the amide I and amide II bands of amide 3.

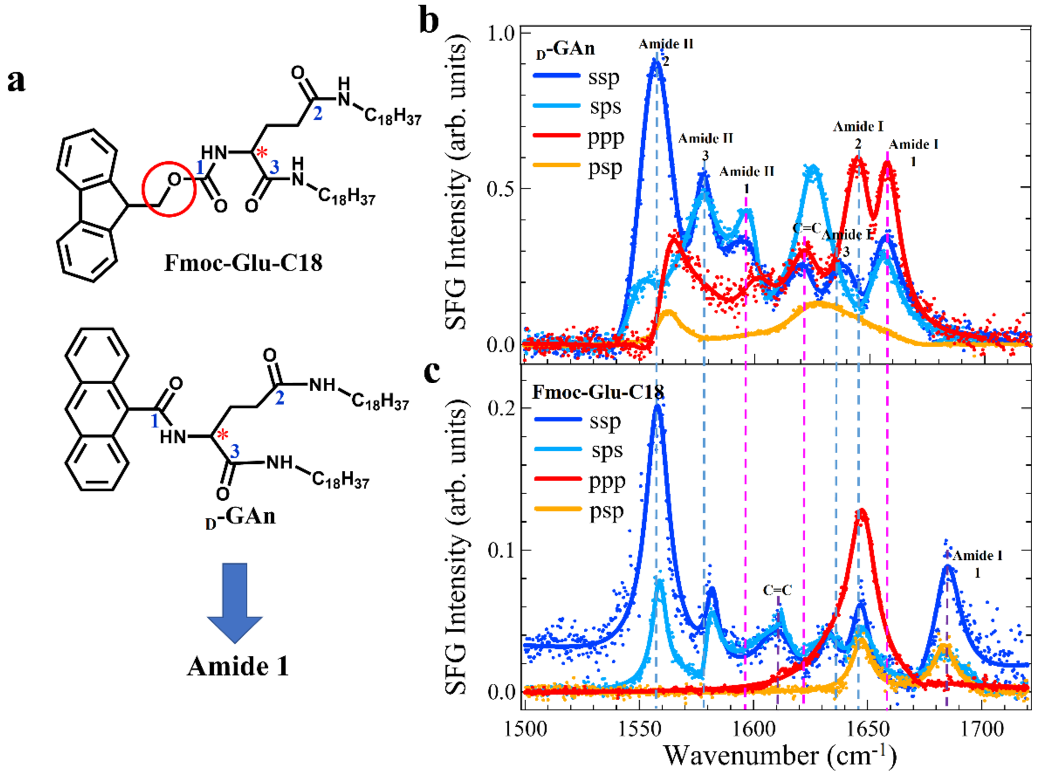

Supplementary Fig. 5 Assign the signal peak of amide 1. a, The molecular structures of Fmoc-Glu-C18 and D-GAn. SFG-VS spectra were recorded using achiral ssp, sps, ppp polarization combinations, and chiral psp polarization combination for (**b**) D-GAn and (**c**) Fmoc-Glu-C18 monolayers (40 mN/m) at the air/water interface. (In Supplementary Fig. 5b, the spectrum measured under the chiral polarization combination used the left-hand coordinate system, and the spectra measured under achiral polarization combinations used the right-hand coordinate system.)

5. L-GAn molecules form an antiparallel *β*-sheet-like structure at the air-water interface

In the above analysis (Figs. 1d and f, Table 1, and Supplementary Section 4), we have clearly assigned three amide groups of L-/D-GAn separately, which allows us to continue to explore the intermolecular hydrogen bonds structures of amide bands. Usually, amide I signal contributed from different secondary motifs in a protein can be separated in the frequency domain using vibrational spectroscopy[19,20]. For example, an α-helix has its characteristic amide I frequency in the range of 1650-1660 cm^{-1}. However, sometimes disordered structure and β-turn may also contribute to amide I signal in this spectral range. Therefore, the assignment of the amide I signal needs to be carefully examined. In this work, we carefully identify the structure of the formed supramolecular assembly not only based on the frequency domain of the chiral peak but also on the relative intensity of the chiral signal and then infer the molecular packing of the molecules self-assemble on the interface.

The symmetry of the repeating unit of an antiparallel β-sheet can be assumed as D_2 symmetry, and theoretically, the B_1, B_2, and B_3 modes are SFG active[21]. The peak at 1637 cm^{-1} (amide I band of amide 3) is attributed to the B_2 mode of the antiparallel β-sheet-like structure, and the B_1 and B_3 modes are not visible in our spectra (Table 1)[22], and the other at 1646 (amide I band of amide 2) and 1657 cm^{-1} (amide I band of amide 1) are attributed to disordered structure and β-turn-like[23]. In order to confirm our inference on the structure, we further explored the polarization-dependent spectroscopy (Supplementary Fig. 6). In which, the achiral and the pure chiral SFG spectra and the chiral spectra obtained by the interference of the two can help us identify the chirality that originates from the specific functional groups (Supplementary Figs. 6a, b, and d). Besides, the intensity of the chiral SFG signal measured by the psp and spp polarization combinations are related to $\chi^{(2)}_{eff,psp}$ and $\chi^{(2)}_{eff,spp}$, respectively, and different secondary structures have different strengths ratios of $\frac{\chi^{(2)}_{eff,psp}}{\chi^{(2)}_{eff,spp}}$ (Supplementary Section 1). Therefore, we can deduce the structure formed by three amide groups of L-/D-GAn

by comparing the strength of the signal in the amide I band under psp and spp chiral polarization combinations with combining the frequency of the signal peak.

In Supplementary Fig. 6c, the chiral signal of amide I of amide 3 (center peak at 1637 cm^{-1}) can be observed under two chiral and two interference chiral polarization combinations, and the chiral SFG vibrational signal are strong. As shown in previous work, antiparallel β-sheet structures can easily pack with twist angles at the interface, therefore generating strong chiral signals for B_1 (1680-1690 cm^{-1}) and B_2 (1630-1640 cm^{-1}) vibrational modes.[24,25] In addition, under the chiral polarization combination of psp and spp, the amide I of amide 3 showed the same intensity, that is $\chi^{(2)}_{eff,psp} = \chi^{(2)}_{eff,spp}$ (Supplementary Table 5), which is consistent with the derived equation ($\chi^{(2)}_{eff,psp} = \chi^{(2)}_{eff,spp} = -\frac{1}{2}N_s(cos^2\theta - sin^2\theta cos^2\psi)\beta_{acb}$) for B_2 mode of antiparallel β-sheet structures in the Supplementary Section 1.4. These results confirmed that the L-/D-GAn formed a significant complex antiparallel β-sheet-like supramolecular chirality structure at the air/water interface. On the contrary, the chiral signal of the amide I of amide 1 and 2 cannot be detected under the spp, s(+m)p-s(-m)p, and p(+m)p-p(-m)p polarization, and the chiral signal of the amide II band are also silent (Supplementary Figs. 6b-d). But it seems to be showed weak chiral spectra features in the amide I band of amide 1 and 2 under the psp polarization combination (Supplementary Fig. 6c). These results indicated that the chiral signals of amide 1 and 2 in the amide I and II regions are very weak and $\chi^{(2)}_{eff,psp} \neq \chi^{(2)}_{eff,spp}$ in amide I region. According to the derived equations (17-21) in Supplementary Section 1.4, there are $\chi^{(2)}_{eff,psp} = \chi^{(2)}_{eff,spp}$ in amide I region for α-helix and β-sheet structures, so we believed amide 1 and 2 forming the disordered structures or non-hydrogen bonded amide group by self-assembly. These disordered structures may have varied symmetry and orientations and contribute to chiral SFG signals that can also behave differently under different polarization combinations.

We also probed the amide III region as an addition to unambiguously identify the supramolecular structure of D-GAn because the characteristic spectral features of α-

helix-like, β-sheet-like, β-turn-like, and random coils are well separated in this spectral region. However, the SFG signal intensity in the amide-III region is so weak, and we did not obtain SFG signals with a reasonable signal-to-noise ratio.

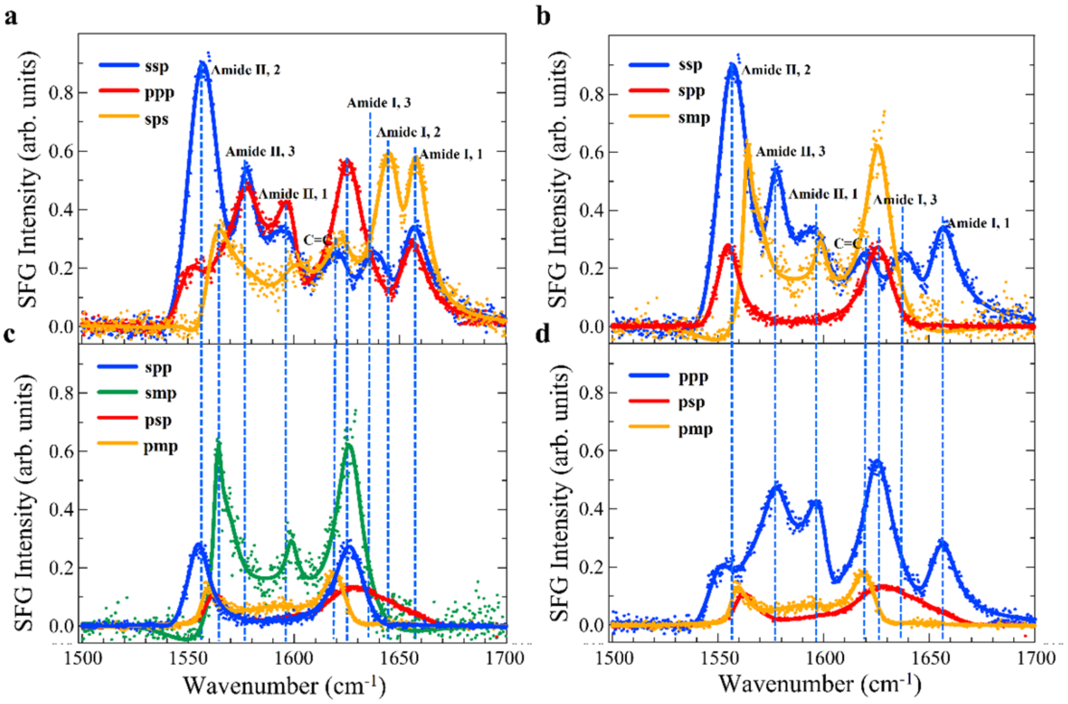

Supplementary Fig. 6 Polarization-dependent detection in the amide band. SFG-VS spectra were recorded using (**a**) achiral ssp, sps, ppp polarization combinations; (**b**) achiral ssp, chiral spp, and their interference chiral s(+m)p-s(-m)p polarization combinations; (**c**) chiral spp, s(+m)p-s(-m)p, psp, and p(+m)p-p(-m)p polarization combinations; (**d**) achiral ppp, chiral psp, and their interference chiral p(+m)p-p(-m)p polarization combinations for D-GAn monolayer in 40 mN/m at the air/water interface.

In this study, L-/D-GAn molecules are not self-assembled into standard secondary structures as proteins or polypeptides because the L-/D-GAn self-assembly at the interface do not have standard peptide chains and are restricted by the interface. However, the L-/D-GAn supramolecular assembly structure still exhibits somewhat antiparallel β-sheet-like structure, because (i) the similar chiral SFG peak position of antiparallel β-sheet-like (amide I band of amide 2-3, 1637 cm^{-1}) is observed, as shown in Supplementary Fig. 6; (ii) the L-/D-GAn assembly shows a regular structure with repeating segments, and the hydrogen bonding pattern exhibits similar properties to that of the β-sheet structure, as shown in Supplementary Fig. 7. It is known that, in the β-sheet conformation, the backbone of the polypeptide chain is extended into a zigzag.

The arrangement of several segments side by side, all of which are in the β conformation, is called a β-sheet. Hydrogen bonds form between adjacent segments of the polypeptide chain within the sheet and are essentially in-line in an antiparallel β-sheet structure. For L-/D-GAn assembly, L-/D-GAn molecules are arranged in columns via intermolecular hydrogen bond and π-π stacking. Each column is formed like a β-sheet, and the hydrogen bonds between the two columns of L-/D-GAn molecules are essentially in-line. The pattern of hydrogen bonds of L-/D-GAn molecules assembled at the interface is very similar to that of antiparallel β-sheet structures in protein or polypeptides. Therefore, we conclude that L-/D-GAn molecules self-assemble at the air/water interface to form antiparallel β-sheet-like supramolecular structures.

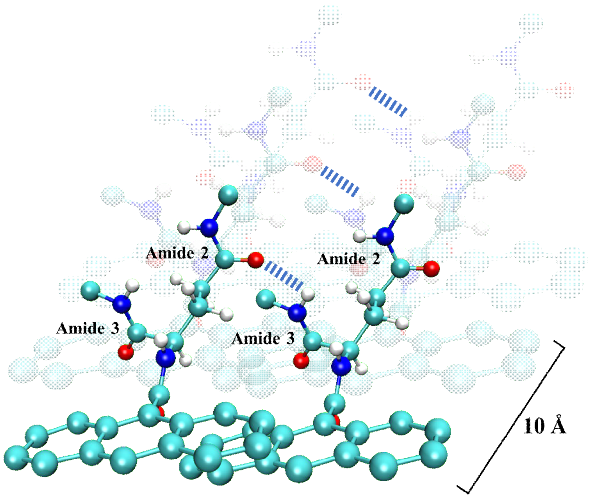

Supplementary Fig. 7 Schematic diagram of hydrogen bond interactions between amide 2 and amide 3 groups of adjacent L-/D-GAn molecules. For the L-/D-GAn assembly with an antiparallel β-sheet-like structure, the repeat period is 10 Å and the hydrogen bond (blue dashed lines) between adjacent L-/D-GAn molecules are essentially in-line.

6. SFG spectra of L-/D-GAn monolayers in different surface pressures

According to the SFG theory in eq.16 ($\chi^{(2)}_{IJK,q} = N\sum_{i,j,k}\langle R_{Ii}R_{Jj}R_{Kk}\rangle\beta^{(2)}_{ijk,q}$), the SFG intensity of any SFG experiment is proportional to the number density of the interface moiety, the orientation angle, and the distribution function. The enhancement of the SFG signals with the surface pressure increases is based on the resultant contribution of the increase in molecular number density and the change in molecular conformation when the surface pressure increases. As reported in the literature[26], the increased ratio

of symmetric stretching vibration intensity of methyl and methylene groups indicates the increase in relative ordering and increases the detected SFG signal (Supplementary Fig. 8b). Such an increased ordering effect for the alkyl chains indicates the L-/D-GAn molecules are more ordered at the higher surface pressure.

Moreover, by comparing the $\frac{\chi_{ssp}}{\chi_{sps}}$ ($\frac{A_{q,ssp}}{A_{q,sps}}$) values of amide I of amide 1 (Supplementary Tables 1-2) for amide groups of L-/D-GAn, we obtained an intensity ratio ($\frac{\chi^{(2)}_{ssp,amide\ I\ of\ amide\ 1}}{\chi^{(2)}_{sps,amide\ I\ of\ amide\ 1}}$) from -0.50 to -0.37 for D-GAn and from -0.50 to -0.41 for L-GAn with the surface pressure increasing. Changes in the intensity ratio of the ssp and sps spectra of the amide I band indicate a change in the conformation of the amide group, which also caused the change in the signal intensity. Therefore, the increase of the SFG signal intensity in the amide and the C-H bands with the surface pressure increases is due to the combined contribution of the increase in molecular number density and the change in molecular conformation.

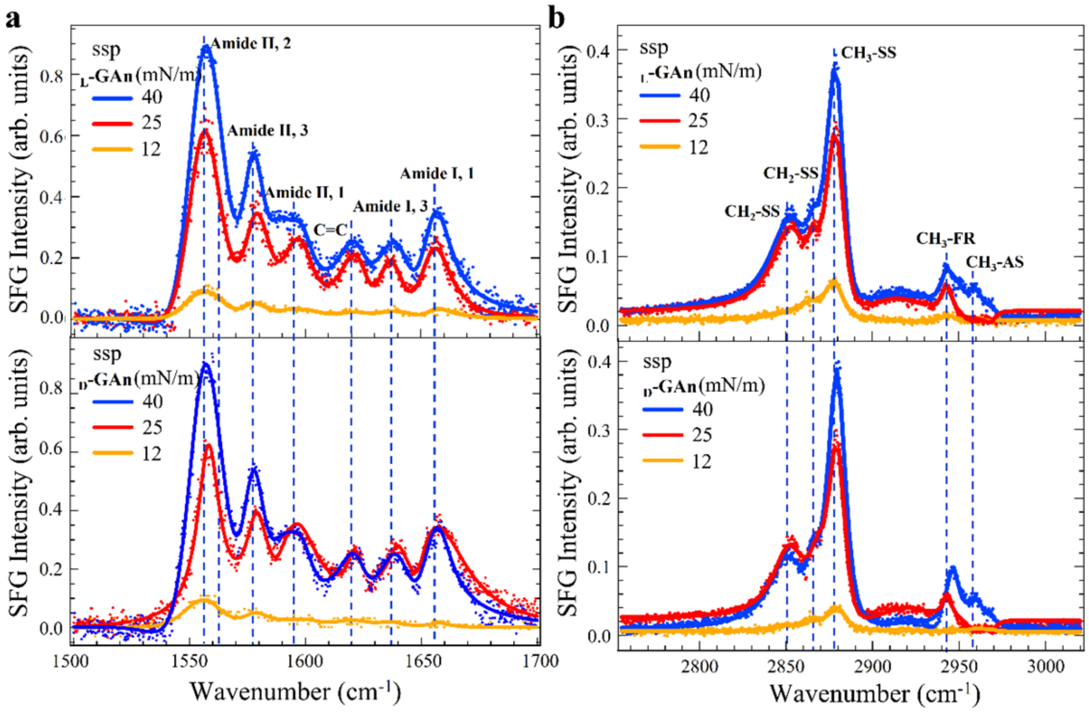

Supplementary Fig. 8 SFG spectra of (**a**) amide modes and (**b**) C-H stretching vibrations of L-GAn and D-GAn monolayers at different surface pressures at the interface.

7. Orientation determination of antiparallel β-sheet-like

SFG spectra collected from amide I modes of peptides and proteins using different polarization combinations can be used to determine membrane orientations of peptides and proteins, as shown in a previous publication.[27] We used polarization-dependent achiral SFG to determine the tilt angle ($\theta,\ \theta \in [0-90°]$) and twist angle ($\psi, \psi \in [0-180°]$) of the β-sheet-like segments of L-/D-GAn molecules. The symmetry of the repeating unit of an antiparallel β-sheet can be treated as D_2 symmetry, and theoretically, the B_1, B_2, and B_3 modes are SFG active.[28] The peak at 1637 cm^{-1} belongs to the B_2 mode of antiparallel β-sheet-like structure, the B_1 and B_3 modes are not observed in our spectral results (Fig. 1d). The nonzero hyperpolarizability tensor elements for B_2 mode we have $\beta_{acb} = \beta_{cab}$.[23] Euler transformations from the molecular coordinates (a, b, c) to laboratory coordinates (X, Y, Z) for an antiparallel β-sheet-like unit were shown in Supplementary Fig. 9. The relationship between the laboratory-fixed axis system and the molecule-fixed coordinate system can be specified by the Euler angles (θ (tilt angle), ψ (twist angle), and φ (in-plane rotation angle)). Euler transformation matrices can be applied to project the microscopic hyperpolarizability from the molecular coordinates to the laboratory coordinates to yield macroscopic second-order susceptibility. In the following discussion, the z-y-z transformation as depicted in the work of the Simpson group is used.[8]

The SFG signals for the ssp, sps, ppp, and psp combinations of the polarization directions are related to $\chi^{(2)}_{IJK}$ tensor elements. For example, the intensity of the ssp achiral SFG signal is related to $\chi^{(2)}_{ssp}$, and consequently $\chi^{(2)}_{YYZ}$. Similarly, the intensity of the chiral SFG signal measured by the psp polarization is related to $\chi^{(2)}_{psp}$ and consequently $\chi^{(2)}_{ZYX}$ in the absence of electronic resonance, details are written in SI. The spectra fitting results are given in Supplementary Tables 1-3. The expressions for these macroscopic susceptibility elements ($\chi^{(2)}_{IJK}$) can be obtained using eq. 4-7. The Euler transformation introduces the molecular orientation (θ, ψ), while the in-plane rotation angle (ϕ) is averaged by the integration over 0 to 2π for an isotropic interface

on the x-y plane to yield. By assuming that the L-/D-GAn monolayers are azimuthally symmetric, we have the following nonzero susceptibility tensor elements for the B_2 modes as follows: [8,23,29]

$$\chi_{ZXY}^{(2)} = -\chi_{ZYX}^{(2)} = -\chi_{YZX}^{(2)} = \chi_{XZY}^{(2)} = \frac{1}{2}N_s(cos^2\theta - sin^2\theta cos^2\psi)\beta_{acb} \quad (24)$$

$$\chi_{YYZ}^{(2)} = N_s(cos^3\theta cos\,\psi\, sin\,\psi - cos\theta\, cos\,\psi\, sin\,\psi)\beta_{acb} \quad (25)$$

$$\chi_{ZZZ}^{(2)} = 2N_s(cos\theta\, cos\,\psi\, sin\,\psi - cos^3\theta\, cos\,\psi\, sin\,\psi)\beta_{acb} \quad (26)$$

$$\chi_{ZYX}^{(2)} = \frac{1}{2}N_s(sin^2\theta cos^2\psi - cos^2\theta)\beta_{acb} \quad (27)$$

$$\chi_{XYZ}^{(2)} = \chi_{YXZ}^{(2)} = 0 \quad (28)$$

In the above equations, N_S is the surface density of the repeating unit of the β-sheet. Assume that the value of hyperpolarizability quantities β_{acb} is -34.5[29], which can be calculated from the Raman tensors and IR transition dipoles. We assumed that both θ and ψ have δ distributions, we can calculate the magnitude of second-order nonlinear susceptibility tensor elements as a function of θ and ψ according to eq. 24-28. From experimental measurements of amide band (Supplementary Tables 1-3), we deduced that the θ and ψ of the antiparallel β-sheet-like structure formed by the L-GAn and D-GAn monolayers under 40 mN/m are 54 ± 0.8° and 50 ± 0.4° (with the ratio $\frac{\chi_{ssp}^{(2)}}{\chi_{sps}^{(2)}} \approx 0.7$ and $\frac{\chi_{ssp}^{(2)}}{\chi_{psp}^{(2)}} \approx -5.9$) and 60 ± 0.3° and 58 ± 0.9° (with the ratio $\frac{\chi_{ssp}^{(2)}}{\chi_{sps}^{(2)}} \approx 0.7$ and $\frac{\chi_{ssp}^{(2)}}{\chi_{psp}^{(2)}} \approx 5.4$), respectively. Due to the low signal-to-noise in the amide band, the fitting numerical tolerances of $\chi^{(2)}$ are relatively large under lower surface pressures, so the orientation angle change under different surface pressures will not be discussed.

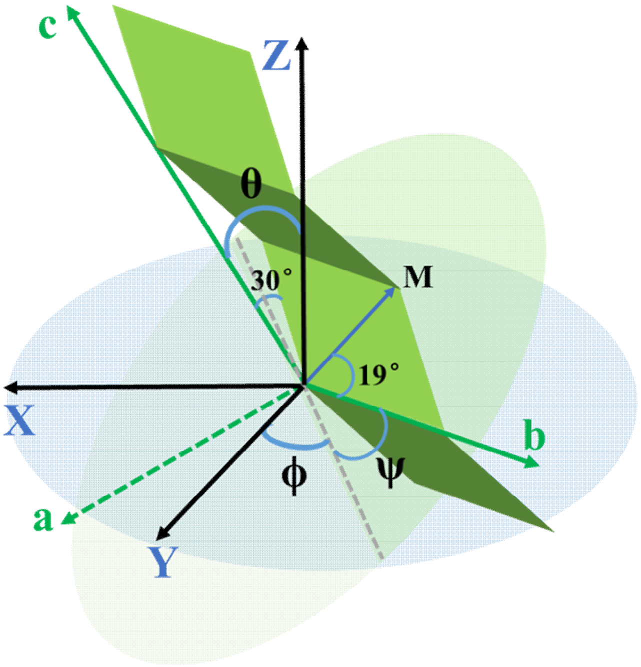

Supplementary Fig. 9 Schematic of amide group orientation. Mutual orientation of the molecule-fixed (a, b, c) and laboratory-fixed (X, Y, Z) axis systems and definitions of the three orientation angles (ϕ, θ, ψ), and the orientation of the IR transition dipole moment of the antiparallel *β*-sheet-like unit formed by L-/D-GAn aggregates at the air/water interface. (The dipole moment of a *β*-sheet-like unit lies in the plane that is inclined at an angle of 30° to the strand axis and makes an angle of 19° to the axis that connects the two successive *α*-carbons of the two strands.)

8. Chiral SFG spectra in the region corresponding to C-H stretching vibrations

As shown in the infrared spectrum of the anthracene molecule, we have observed the =C-H stretching vibration peak of the anthracene ring at 3046 cm^{-1} (Supplementary Fig. 10a). However, in the SFG measurement at 2750-3150 cm^{-1}, both the chiral SFG signal and achiral SFG signal of the =C-H stretching vibrations of the anthracene groups of L-GAn and D-GAn molecules (center peak at 3046 cm^{-1}) cannot be observed (Supplementary Figs. 10c and e) even the IR laser intensity at 3000-3100 cm^{-1} is high enough (Supplementary Fig. 10b). Supplementary Fig. 10b shows the non-resonant SFG signal from z-cut quartz, from which we conclude that the IR laser intensity is high enough to get reasonable SFG spectra for =C-H stretching vibrations of the anthracene groups. The absence of the =C-H SFG signal might have two reasons. One is that the anthracene ring has a centrosymmetric structure that the dipole moment of the =C-H has been canceled out. The second reason is the anthracene rings parallel to

the interface; which can be confirmed by MD simulation (about 70° with an orientation contribution of 0-160° (Fig. 3f).) Moreover, the weak resonant SHG signal on the D-GAn monolayer which indicated the anthracene ring should lie on the interface because the typical absorption of anthracene is in the 330-400 nm when the incident laser beam is 780 nm (Supplementary Fig. 10d).

It should be noted that although the chiral SFG response of aromatic =C-H stretches is not present, we can still conclude that the chirality transfer from the chiral-center carbon to the anthracene group from CD spectra and SHG spectra. The CD spectra of 40-layer Langmuir-Schaefer films of L-GAn and D-GAn show both the ^1B_b- and ^1L_a-bands of the anthracene of L-GAn and D-GAn show mirror-symmetric CD signals[30]. And the chiral SHG spectra of the D-GAn and L-GAn monolayer at the air/water interface shows opposite chiral signals (Supplementary Fig. 10f). Moreover, we also detected mirror image chiral SFG signals of C=C stretching vibration peaks in the anthracene groups of L-GAn and D-GAn molecules in the amide band (Figs. 1e and g). These results indicate that during the interfacial assembly of L-GAn and D-GAn molecules, their aromatic groups exhibit opposite chiral characteristics by asymmetric stacking and twisting induced by chiral centers.

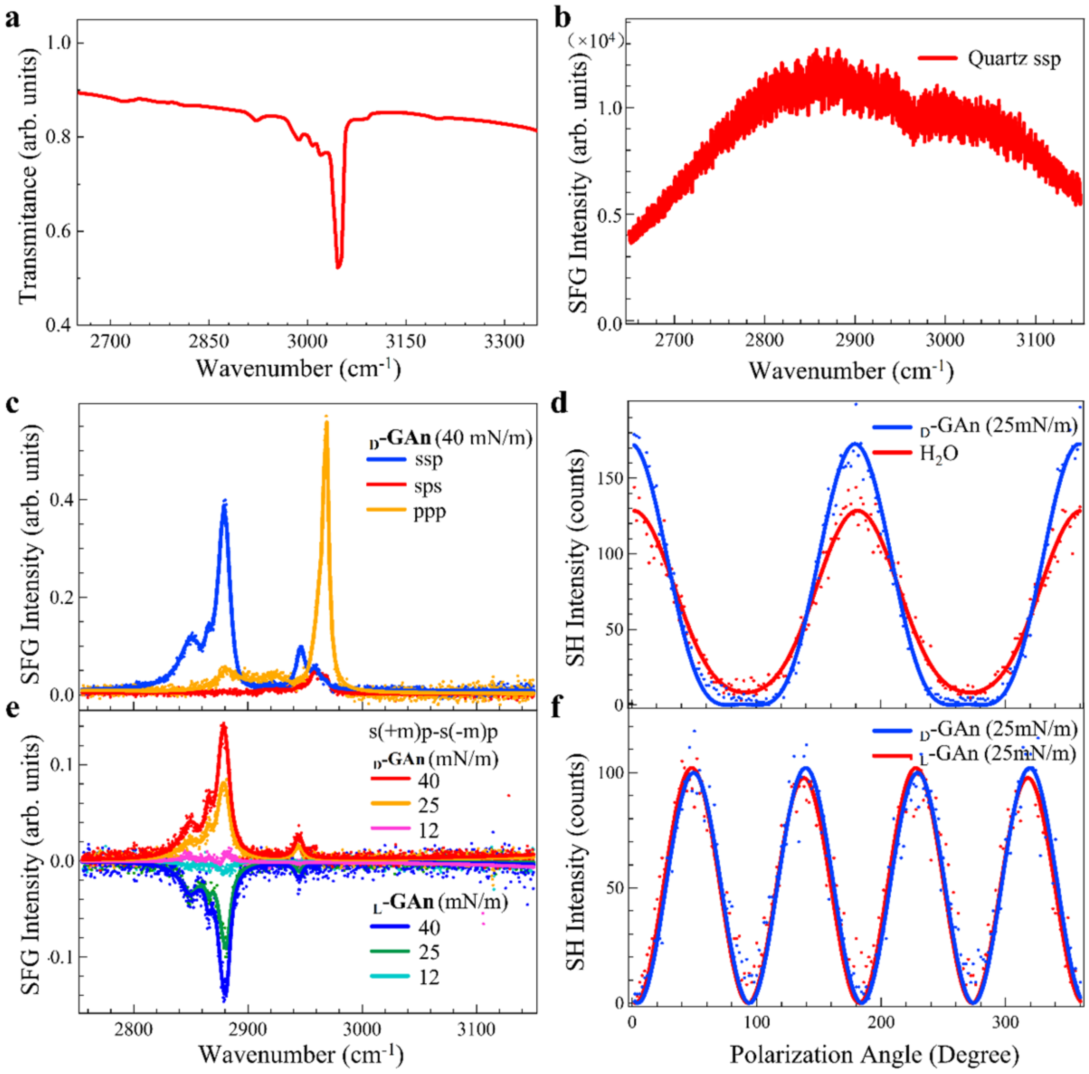

Supplementary Fig. 10 Aromatic =C-H stretching vibration spectra of anthracene groups of D-GAn molecules. a, Fourier transform infrared spectroscopy (FTIR) of anthracene molecules (KBr pellet method). **b**, SFG spectra of the z-cut quartz under ssp polarization. **c**, Achiral SFG spectra of the D-GAn monolayer at a surface pressure of 40 mN/m in the C-H band at the air/water interface. **d**, P-polarization detection of the 390 nm SHG signal from the D-GAn monolayer and pure H_2O at the air/water interface. **e,** Chiral SFG spectra of the L-GAn and D-GAn monolayers at different surface pressures at the air/water interface. **f**, The s-polarization detection of the 390 nm SHG signal from the D-GAn and L-GAn monolayer at the air/water interface ($DCE = \frac{\Delta I}{I} = \frac{2(I_{+45°s} - I_{+135°s})}{(I_{+45°s} + I_{+135°s})}$, the DCE values of the D-GAn and L-GAn monolayer are -0.02 and 0.03, respectively).

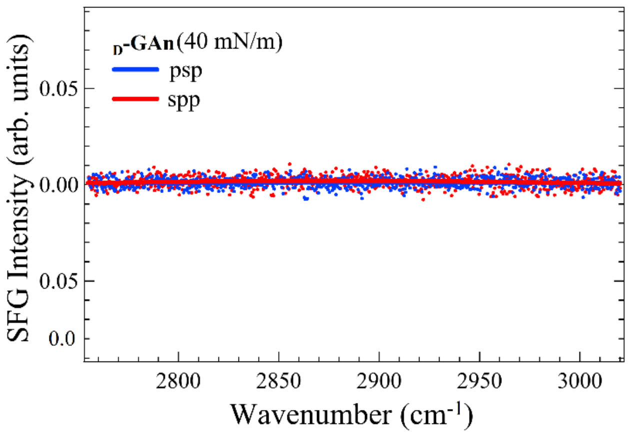

Supplementary Fig. 11 Chiral SFG spectra in the region corresponding to C-H stretching vibrations. SFG-VS spectra were recorded using pure chiral psp, spp polarization combination for D-GAn monolayer at a surface pressure of 40 mN/m at the air/water interface.

9. Orientation determination of methyl groups

The macroscopic chirality response on surfaces detected by SFG spectra comes from both the intrinsic chirality of chiral molecules and the orientational or structural chirality. The contribution of the achiral or chiral CH_3 groups to the chiral SFG spectra is orientational or structural chirality, as the result of averaging over the orientational distributions of the surface molecular groups.[31] Here, we measure the orientations of the methyl group and amide group separately and then deduce the conformation and overall orientation of the molecules. The chiral information on the carbon atom in the chiral center is already very weak when it passes through the 18-carbon alkyl chains to the terminal methyl groups and cannot be detected with the independent chiral polarization. Therefore, we assumed that the end methyl groups of the alkyl chain obey C_s symmetry, and used the ratio method widely reported in the literature to determine molecular group orientation angles at the interface.[32-34] Experimentally, the CH_3 vibrational peaks in the SFG-VS ssp and ppp spectra well obey the polarization selection rules for the end methyl groups as shown in the ssp and ppp SFG spectra, as in Fig. 2. The effective SFG susceptibility $\chi_{eff}^{(2)}$ can be simplified to the following expression:[35]

$$\chi_{eff}^{(2)} = N_S \cdot d \cdot r(\theta) \tag{29}$$

Where N_S is the surface density of the probed interfacial species; d is the susceptibility strength factor, which is a constant in a specific experimental configuration for a given molecular system; and $r(\theta)$ is the orientational field functional, which contains all orientational information at a given SFG experimental configuration.[28,36] The equation shows that $\chi_{eff}^{(2)}(A_q)$ is a function of interfacial molecular density N_S and orientation angle (θ). The sps intensity in the whole orientation angle (θ) range was close to zero at C-H bands, therefore using the ratio $\frac{A_{q,ssp}}{A_{q,ppp}}$ to simulated orientation angle of CH_3 groups in this work.

With the global fitting results as listed in Supplementary Tables 6-8, a detailed analysis of the orientation of the CH_3 groups in the L-/D-GAn Langmuir monolayers can be carried out. SFG orientation analysis of surface/interfacial methyl groups also has been studied extensively and a C_s symmetry can be approximately used for a methyl group at the end of the alkyl chain, in such SFG simulation using the bond polarizability model.[37] When analyzing the orientation of the methyl group of L-/D-GAn, we used the Raman tensor ratio r of the DPPC methyl group reported in the literature, that is, $\frac{r=\beta_{aac}}{\beta_{ccc}} = 4.0$,[38] because these molecules have long alkyl chains and similar spectral characteristics in the C-H stretch region. We modeled the $\chi_{eff}^{(2)}(A_q)$ versus θ curves for C_s symmetry (Supplementary Fig. 12) according to eq. 29, this orientation angle value is directly proportional to the A_q value as listed in Supplementary Tables 6 and 7.

It is interesting to note that there are approximative global-fitting results of the achiral SFG spectra of L-/D-GAn monolayers in Supplementary Tables 6 and 7, which suggested that the corresponding alkyl chains in the L-GAn and D-GAn enantiomers are with the same interfacial orientations with respect to the interface normal. By comparing this experimental $\frac{\chi_{ssp}}{\chi_{ppp}}(\frac{A_{q,ssp}}{A_{q,ppp}})$ values with the theoretically simulated values as a δ-function of the curves in Supplementary Fig. 12 (purple line), we obtained θ from about 44° (with $\frac{\chi_{ssp,L-GAn}^{(2)}}{\chi_{ppp,L-GAn}^{(2)}} \approx 39.0$, $\frac{\chi_{ssp,D-GAn}^{(2)}}{\chi_{ppp,D-GAn}^{(2)}} \approx 35.0$) to 37° (with $\frac{\chi_{ssp,L-GAn}^{(2)}}{\chi_{ppp,L-GAn}^{(2)}} \approx -10.6$,

$\frac{\chi^{(2)}_{ssp,D-GAn}}{\chi^{(2)}_{ppp,D-GAn}} \approx -9.7$) for the methyl group of the interfacial L-GAn and D-GAn molecules with the surface pressure rising. That means with the increase of surface pressure, the alkyl chains stand straighter and molecules self-assembly forming a more orderly assembly structure, which is consistent with the changes of morphology and SFG intensity in the C-H stretch region.

As discussed above, for the L-GAn monolayer constructed at the air-water interface, we used the ssp and ppp polarization intensity ratio to calculate the orientation angles of the CH_3 groups at different surface pressure and we found the tilt angle was changed from 44° to 37° which is very close to the magic angle (Supplementary Fig. 12). But this magic angle should not affect the main conclusions drawn from HR-SFG-VS (0.4 cm^{-1}) orientational analysis. The reasons are as follows. Firstly, the narrow spectral feature centered at 2880 cm^{-1} (FWHM is 8.6 cm^{-1}, as shown in Supplementary Table 6) suggests that the CH_3 group is quite ordered at the interface, which indicates the σ value of the methyl groups at the interface is limited. Secondly, the change of the D value of the CH_3 group at two surface pressure are also an indication that the σ value of the CH_3 group is limited. If the σ value is broad the D value would not change. Third, as the analysis in the literature for free O-H bond of water molecules at the topmost layer suggest, even one assume the relatively large σ as 15°, the allowed θ_0 values corresponding to the magic angle θ = 39.2° can vary only less than 3° from σ = 15° to σ = 0°[39]. For the amphiphilic molecule at the interface, the dynamic of the CH_3 group located at the long alky chain are not fast like air/water interface, and the standard deviation σ of CH_3 should be smaller than the free OH.

In Supplementary Fig. 12, we use the mathematic simulated ssp/ppp ratio for the CH_3-SS peak to calculate the orientational tilt angle. The horizontal dash line indicates the ratio from experimental fittings, and the vertical dashed lines give the range of the orientational angle of the CH_3- group with the Gaussian distribution of σ = 15° and σ = 0°. With σ < 15°, the allowed θ_0 values corresponding to the tilt angle θ = 37° can vary from 34 to 37°, that is only about 3° from σ = 15° to σ = 0°. The tilt angle θ = 44° can vary from 43 to 44, that is only about 1° from σ = 15° to σ = 0°. Therefore, the existence

of the SFG-VS magic angle should not affect the main conclusions drawn from our HR-SFG-VS orientational analysis, the spectra provided the orientational parameter D was determined with enough accuracy.

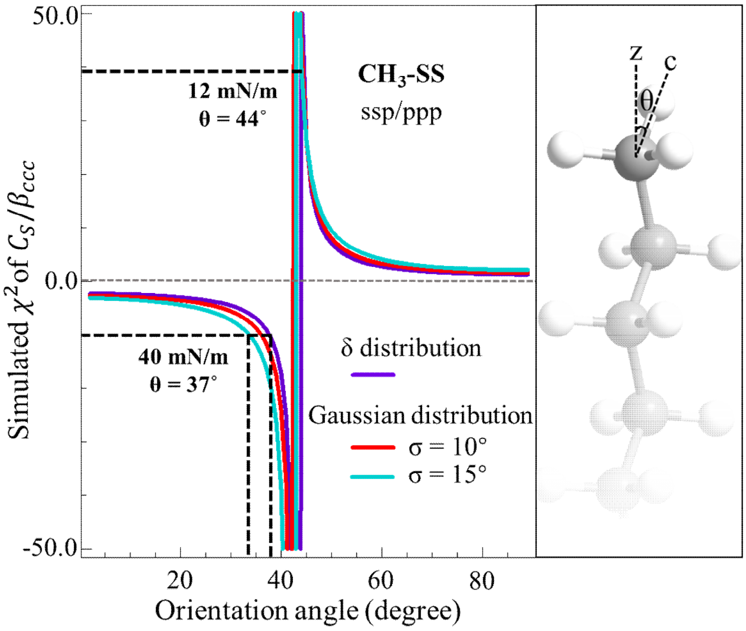

Supplementary Fig. 12 The apparent molecular tilt angle (calculated by incorrectly assuming a δ-function distribution, as in eq $D \equiv \frac{\langle cos^3\theta \rangle}{\langle cos\theta \rangle} = \frac{\chi^{(2)}_{zzz}}{\chi^{(2)}_{zzz}+2\chi^{(2)}_{zxx}} \cong cos^2\langle\theta\rangle$) as a function of the root-mean-square width (σ) of a Gaussian distribution. The ratio of symmetric stretching vibration intensity under ssp and ppp polarization for CH_3 group vs the orientation angle θ under the δ distribution and Gaussian distribution (σ=10° and σ=15°). The purple line is under the δ distribution. The red and blue lines are under Gaussian distribution. The horizontal dash line indicates the ratio from experimental fittings, the vertical dash line indicates orientation angle of the CH_3 group at different orientation distribution. The illustration on the right shows a methyl group unit of the alkyl chain and the orientation angle of the methyl group in the given molecular frame. Axis c is the principal axis of the methyl group, axis z represents the surface normal, orientation angle (θ) is defined as the angle between axis z and axis c.

10. Molecular snapshots of self-assembly

The initial state of the simulated system (L-GAn monolayer spread at the air/water interface) is presented by t = 0 s. Under 1 bar of lateral pressure, and limiting the orientation of the anthracene ring to remain unchanged, after 5 ns, L-GAn forms a dense monolayer on the interface with a monomolecular area of 53 Å^2 (the surface pressure is about 20 mN/m, as shown in Supplementary Fig. 2). After 55 ns, it is observed that the anthracene ring of the molecule has undergone a larger twist, and the lateral

interaction between the molecules has been strengthened, as shown in Supplementary Fig. 13a.

The top-view and side-view system snapshots of the last frame as shown in Supplementary Figs. 13b-c. The simulation system consists of 100 L-GAn molecules (10×10 molecules) and many water molecules. In order to ascertain the assembly structure of the molecules, we selected 9 molecules (red box) and 4 molecules (purple box) from the central area of the box for coloring in the VMD, as shown in the main text of Figs. 3b-c.

As mentioned above, under the orientation restriction of the molecular head group was released and the lateral pressure was no longer applied, a 50 ns simulation was performed again. The total number of hydrogen bonds formed between L-GAn molecules was counted as a function of the simulation time in Supplementary Fig. 13d.

The probability statistics of the height of a single-molecule after 55 ns dynamics simulation are shown in Supplementary Fig. 13e. The result showed that the height of the single-molecule is between 33-41 Å^2 and deduced that the thickness of the monolayer is about 36 Å^2.

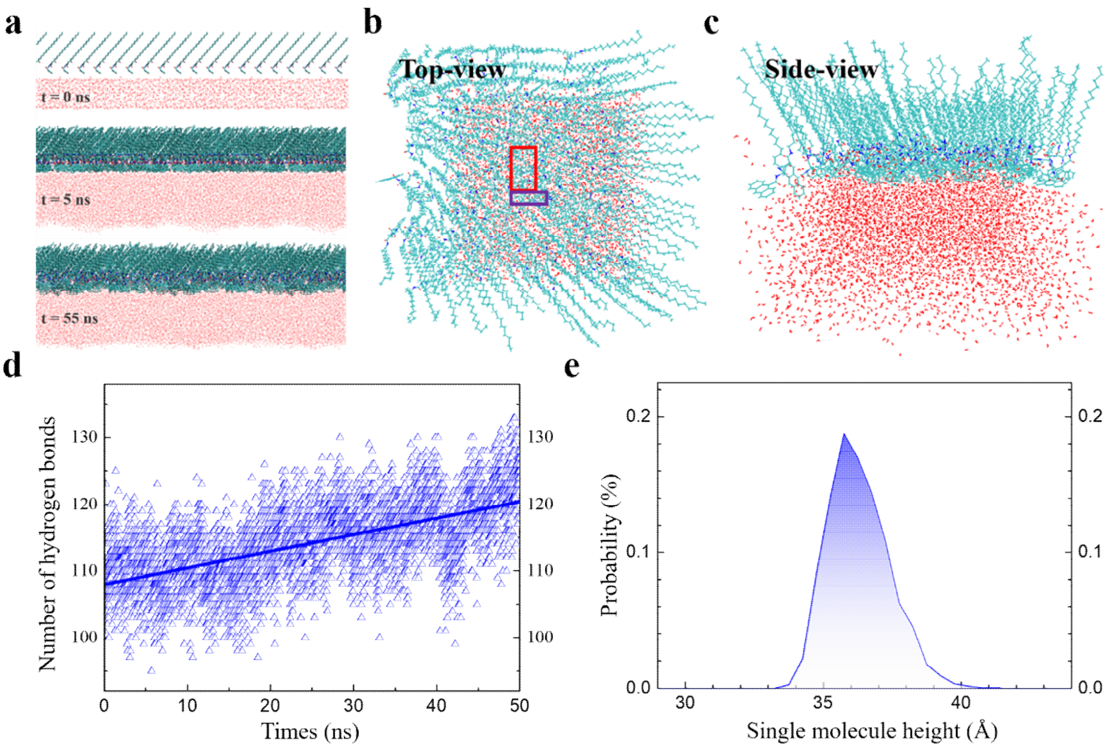

Supplementary Fig. 13 The results of MD simulations. a, Side-view system snapshots for the

self-assembly process of L-GAn molecules at the air/water interface. The (**b**) top-view and (**c**) side-view system snapshots of the last frame after 55 ns MD simulation. The red and purple boxes indicated the molecular positions shown in Figs. 3b-c of the main text, respectively. **d,** The number of hydrogen bonds between L-GAn molecules changes with the simulation time. The solid line is the result of the linear fitting. **e,** After performing a 55 ns simulation, the probability statistics of the height of a single molecule.

11. Number of molecules assembled into nanorods

The monolayers were transferred onto the freshly cleaved mica surface and their atomic force microscopy (AFM) was measured. As reported in published references, for the L-GAn Langmuir-Blodgett films at 15 mN/m, nanorods with a height of 29-43 Å^2 were observed (Supplementary Figs. 14a and b), and when the surface pressure was increased to 30 mN/m, the nanorods were packed closer[30].

We calculated the distance between the alkyl chain end methyl and the anthracene rings of the 100 L-GAn molecules that reached equilibrium after 55 ns MD simulation; the highest probability is located at 36 Å (Supplementary Fig. 13e). We deduced that the thickness of the monolayer is about 36 Å. The length and width of a single L-GAn molecule at the air/water interface are also calculated by MD simulation. Since molecules located at box boundaries tend not to have ordered orientations, we measured the length (9.2 Å) and width (5.0 Å) of several molecules in the central region highlighted in Figs. 3b (bottom) and c (bottom) to estimate the molecular size of the self-assembly into nanorods accurately (Supplementary Figs. 14 c-d). We choose the longest nanorod measured by AFM (length 4560 Å and width 250 Å), and the L-GAn molecules at the interface are driven by π-π interaction to build the length of the nanorods along the y-axis direction and driven by the hydrogen bond to build the width of the nanorods along the x-axis direction. The length, width, and height of the L-GAn nanorods can be estimated to be 912 (4560 Å/5.0 Å, y-axis), 27 (250 Å/9.2 Å, x-axis) and single molecules (z-axis), respectively.

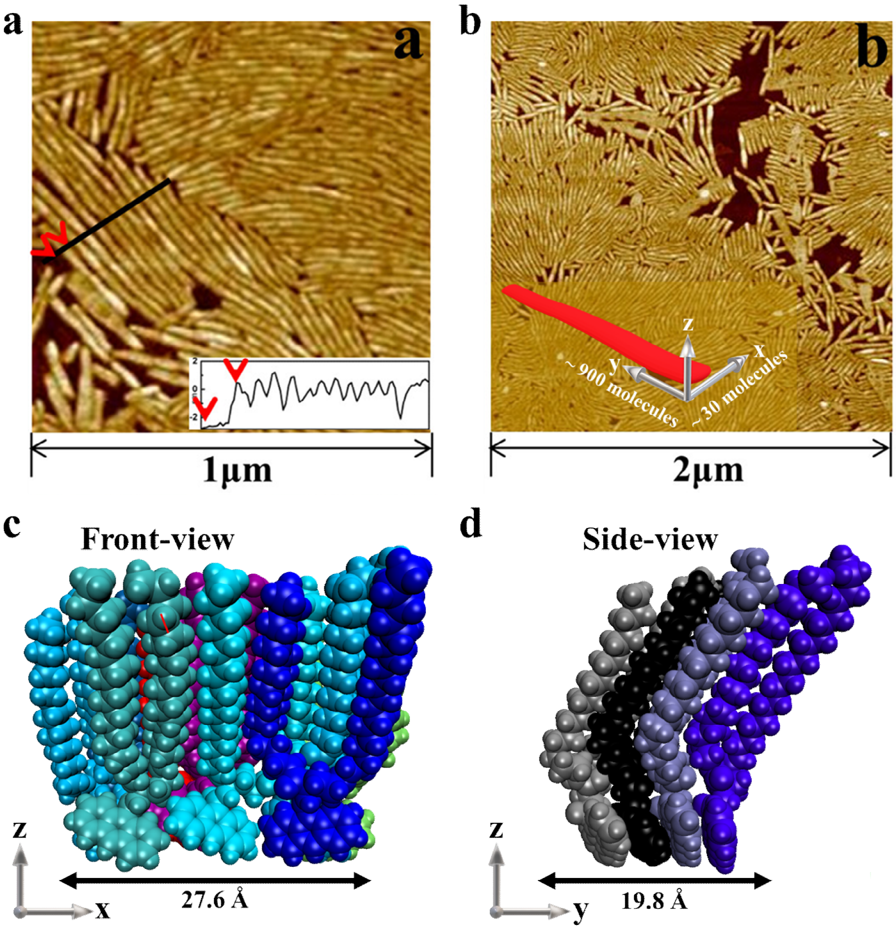

Supplementary Fig. 14 AFM image of L-GAn monolayer and the snapshots of L-GAn molecules at 55 ns. a-b, AFM images of one-layer Langmuir-Blodgett film of L-GAn deposited onto a freshly cleaved mica surface from the water subphase at 15 mN/m. The room temperature is 25°C. Adapted with permission from Supplementary References [30]. Copyright [2019] Langmuir. The inset at the bottom of Supplementary Fig. 14b is an illustration of the nanorods in the three-dimensional coordinate orientation. The length (4560 Å), width (250 Å), and height (25 Å) of the nanorod are composed of 912 molecules, 27 molecules, and single molecule, respectively. **c**, Front-view and (**d**) side-view of several L-GAn molecules located in the central region of the box after 55 ns MD simulation.

As shown in Supplementary Figs. 15a-c, Fmoc-Glu-C18 molecules self-assemble at the air-water interface to form nanorod structures with a height of about 25 Å at a surface pressure of 15 mN/m. When the surface pressure is increased to 25 mN/m, the nanorods are more densely packed, and the defects of the monolayer formed by stacking many nanorods are significantly reduced (Supplementary Figs. 15d).

We now attempt to characterize the Fmoc-Glu-C18 supramolecular self-assembly from AFM imagines using the same method as the L-GAn supramolecular self-assembly. We assume that L-GAn and Fmoc-Glu-C18 assemblies have similar three-dimension

single-molecule sizes at the interface because the molecular structures of Fmoc-Glu-C18 molecules and L-GAn molecules are similar (Fig. 1a), which of both self-assembly form nanorod structures with similar shapes (Supplementary Figs. 14a-b and Supplementary Fig. 15). Using the AFM results in Supplementary Fig. 15 and MD simulation of the L-GAn assemblies, we can roughly estimate the number of Fmoc-Glu-C18 molecules constructed a single nanorod. The Fmoc-Glu-C18 molecules at the interface are driven by π-π interaction to build the length of the nanorods along the y-direction of the coordinate axis and driven by the hydrogen bond to build the width of the nanorods along the x-direction of the coordinate axis. The length, width, and height of the nanorods can be estimated to be 400 molecules (2187Å/5.0Å, y-axis), 40 molecules (375Å/9.2Å, x-axis), and a single molecule (z-axis), respectively (Supplementary Fig. 15d). It must be pointed out that the number of Fmoc-Glu-C18 molecules assembled to form nanorods in the xyz direction above is just a rough estimate. In the future, we will calculate the length, width, and height of a single Fmoc-Glu-C18 molecule to form supramolecular assemblies at the interface by MD simulation to gain the exact number of molecules of the nanorods.

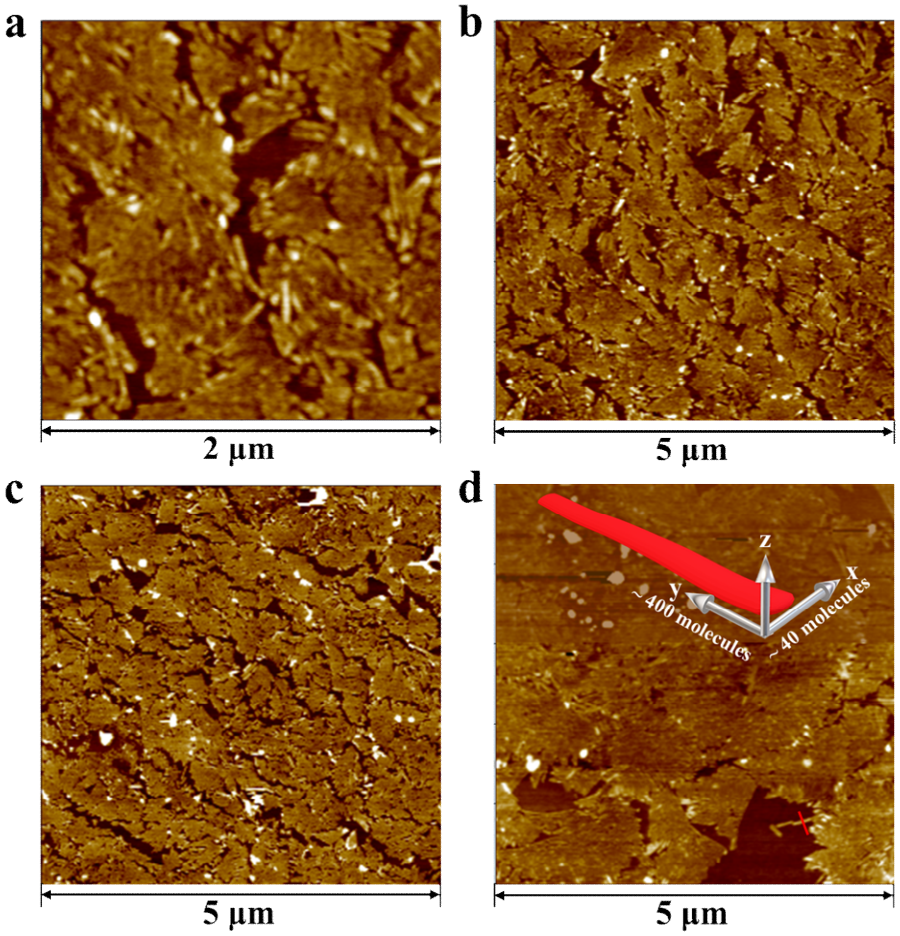

Supplementary Fig. 15 AFM image of Fmoc-Glu-C18 monolayer. AFM images of one-layer Langmuir-Blodgett film of Fmoc-Glu-C18 deposited onto a freshly cleaved mica surface from the water subphase at (**a-c**) 15 mN/m and (**d**) 25 mN/m. The room temperature is 25°C. The upper inset of Supplementary Fig. 15d is an illustration of the nanorod in the three-dimensional coordinate direction. The length (2187 Å), width (375 Å), and height (25 Å) of the nanorod are composed of 400 molecules, 40 molecules, and single molecule, respectively.

12. Global fitting of SFG spectra in amide region

Supplementary Table 1 The Global-Fitting parameters and vibrational mode assignments for the spectra of L-GAn monolayer in the amide region. The spectra are fitted with Lorentzian line shape function as Eq. 2. The peak position of the vibrational modes ω_q, the peak width Γ_q, and the oscillator strength factor $\chi_{\mathrm{eff},q,ijk}$ (A_{ssp}, A_{sps}, A_{ppp}) of the vibrational modes and assignment of the characteristic peaks are listed. The data in Supplementary Table 1 is used to calculate the orientation angle of the amide group of L-GAn.

Surface pressure (mN/m)	ω_q (cm^{-1})	Γ_q (cm^{-1})	A_{ssp} (a.u.)	A_{sps} (a.u.)	A_{ppp} (a.u.)	Assignment
40	1550.8 ± 0.8	14.1 ± 1.4	-3.9 ± 1.3	7.3 ± 2.5	17.7 ± 3.3	-
	1554.2 ± 2.4	14.8 ± 0.8	31.2 ± 1.1	12.0 ± 1.7	9.1 ± 1.2	Amide II, 2
	1562.8 ± 0.4	8.7 ± 0.8	2.2 ± 0.9	4.8 ± 1.3	0.8 ± 0.3	-
	1577.6 ± 2.1	7.5 ± 1.2	6.6 ± 0.3	0.0 ± 0.3	1.9 ± 0.6	Amide II, 3
	1598.3 ± 0.2	7.6 ± 0.3	1.8 ± 0.3	0.4 ± 0.0	3.2 ± 0.2	Amide II, 1
	1603.0 ± 1.0	6.4 ± 1.5	-0.2 ± 0.1	0.5 ± 0.3	-1.6 ± 0.5	-
	1621.4 ± 1.1	10.4 ± 0.1	8.6 ± 2.5	5.8 ± 0.5	3.9 ± 0.1	C= C (Anthracene)
	1624.8 ± 0.1	9.2 ± 1.2	-1.4 ± 0.8	-0.5 ± 0.8	2.0 ± 0.8	-
	1637.5 ± 3.1	14.7 ± 2.1	8.3 ± 1.4	12.5 ± 3.0	1.3 ± 0.2	Amide I, 3
	1646.9 ± 0.5	11.3 ± 0.2	2.2 ± 1.0	17.2 ± 1.0	-2.6 ± 1.0	Amide I, 2
	1656.7 ± 0.1	7.7 ± 1.1	-2.9 ± 0.6	7.1 ± 0.9	10.4 ± 0.6	Amide I, 1
25	1550.8 ± 0.8	14.1 ± 1.4	-20.5 ± 3.8	3.4 ± 1.4	-0.1 ± 0.4	-
	1554.2 ± 2.4	14.8 ± 0.8	31.1 ± 4.7	-6.3 ± 1.2	2.7 ± 1.1	Amide II, 2
	1562.8 ± 0.4	8.7 ± 0.8	1.3 ± 0.5	1.6 ± 1.3	0.5 ± 0.3	-
	1577.6 ± 2.1	7.5 ± 1.2	4.5 ± 0.2	-1.2 ± 0.5	2.4 ± 0.8	Amide II, 3
	1598.3 ± 0.2	7.6 ± 0.3	1.1 ± 0.8	0.0 ± 0.1	1.7 ± 0.6	Amide II, 1
	1603.0 ± 1.0	6.4 ± 1.5	0.6 ± 0.1	0.2 ± 0.1	-0.5 ± 0.2	-
	1621.4 ± 1.1	10.4 ± 0.1	7.6 ± 2.5	4.4 ± 0.5	2.9 ± 0.2	C= C (Anthracene)
	1624.8 ± 0.1	9.2 ± 1.2	1.3 ± 0.9	-0.2 ± 0.0	2.3 ± 0.9	-
	1637.5 ± 3.1	14.7 ± 2.1	2.1 ± 0.3	3.0 ± 0.1	0.4 ± 0.2	Amide I, 3
	1646.9 ± 0.5	11.3 ± 0.2	1.8 ± 0.6	2.6 ± 0.8	-0.4 ± 0.0	Amide I, 2
	1656.7 ± 0.1	7.7 ± 1.1	-1.3 ± 0.3	0.9 ± 0.1	1.3 ± 0.2	Amide I, 1
12	1550.8 ± 0.8	14.1 ± 1.4	0.2 ± 0.1	-0.3 ± 0.2	0.8 ± 0.5	-
	1554.2 ± 2.4	14.8 ± 0.8	2.2 ± 0.0	0.1 ± 0.0	0.1 ± 0.0	Amide II, 2
	1562.8 ± 0.4	8.7 ± 0.8	0.1 ± 0.0	0.2 ± 0.0	0.0 ± 0.0	-
	1577.6 ± 2.1	7.5 ± 1.2	1.1 ± 0.4	0.1 ± 0.2	0.4 ± 0.3	Amide II, 3
	1598.3 ± 0.2	7.6 ± 0.3	0.2 ± 0.1	0.0 ± 0.1	0.3 ± 0.1	Amide II, 1
	1603.0 ± 1.0	6.4 ± 1.5	0.1 ± 0.1	0.0 ± 0.0	-0.0 ± 0.1	-
	1621.4 ± 1.1	10.4 ± 0.1	0.9 ± 0.4	0.2 ± 0.4	0.6 ± 0.5	C= C (Anthracene)
	1624.8 ± 0.1	9.2 ± 1.2	-0.6 ± 0.1	-0.3 ± 0.0	-0.1 ± 0.1	-
	1637.5 ± 3.1	14.7 ± 2.1	0.6 ± 0.1	0.9 ± 0.1	0.2 ± 0.1	Amide I, 3
	1646.9 ± 0.5	11.3 ± 0.2	0.5 ± 0.1	1.1 ± 0.1	-0.3 ± 0.1	Amide I, 2
	1656.7 ± 0.1	7.7 ± 1.1	-0.2 ± 0.1	0.4 ± 0.0	0.1 ± 0.0	Amide I, 1

Supplementary Table 2 The Global-Fitting parameters and vibrational mode assignments for the spectra of the D-GAn monolayer in Fig. 1d. The Global-Fitting results of the SFG-VS spectra of D-GAn monolayer in different surface pressures at the amide region. The spectra are fitted with Lorentzian line shape function as Eq. 2. The peak position of the vibrational modes ω_q, the peak width Γ_q, and the oscillator

strength factor $\chi_{\text{eff},q,ijk}$ (A_{ssp}, A_{sps}, A_{ppp}) of the vibrational modes and assignment of the characteristic peaks are listed. The data in Supplementary Table 2 is used to calculate the orientation angles of the amide group of D-GAn.

Surface pressure (mN/m)	ω_q (cm^{-1})	Γ_q (cm^{-1})	A_{ssp} (a.u.)	A_{sps} (a.u.)	A_{ppp} (a.u.)	Assignment
	1550.8 ± 0.8	14.1 ± 1.4	-1.5 ± 0.1	6.8 ± 2.2	18.5 ± 4.4	-
	1554.2 ± 2.4	14.8 ± 0.8	28.9 ± 2.9	-11.6 ± 3.4	9.5 ± 4.8	Amide II, 2
	1562.8 ± 0.4	8.7 ± 0.8	2.0 ± 0.1	4.7 ± 1.3	0.7 ± 0.7	-
	1577.6 ± 2.1	7.5 ± 1.2	6.3 ± 1.7	0.0 ± 0.0	1.8 ± 0.4	Amide II, 3
	1598.3 ± 0.2	7.6 ± 0.3	2.0 ± 0.7	0.5 ± 0.2	3.2 ± 1.5	Amide II, 1
40	1603.0 ± 1.0	6.4 ± 1.5	-0.1 ± 0.4	0.4 ± 0.1	-1.6 ± 1.3	-
	1621.4 ± 1.1	10.4 ± 0.1	7.6 ± 0.0	5.9 ± 0.3	4.1 ± 0.3	C= C (Anthracene)
	1624.8 ± 0.1	9.2 ± 1.2	-1.7 ± 1.0	-1.3 ± 0.8	1.6 ± 0.8	-
	1637.5 ± 3.1	14.7 ± 2.1	8.8 ± 1.3	12.9 ± 1.3	1.2 ± 0.6	Amide I, 3
	1646.9 ± 0.5	11.3 ± 0.2	1.9 ± 0.8	19.3 ± 3.2	2.8 ± 0.5	Amide I, 2
	1656.7 ± 0.1	7.7 ± 1.1	-2.6 ± 0.6	7.0 ± 1.1	9.4 ± 1.4	Amide I, 1
	1550.8 ± 0.8	14.1 ± 1.4	-1.9 ± 1.1	5.6 ± 1.1	-6.1 ± 1.1	-
	1554.2 ± 2.4	14.8 ± 0.8	22.1 ± 3.7	-7.5 ± 1.0	7.6 ± 1.0	Amide II, 2
	1562.8 ± 0.4	8.7 ± 0.8	1.2 ± 0.5	1.8 ± 0.3	0.5 ± 0.2	-
	1577.6 ± 2.1	7.5 ± 1.2	5.9 ± 0.7	-1.5 ± 0.2	4.3 ± 0.4	Amide II, 3
	1598.3 ± 0.2	7.6 ± 0.3	1.9 ± 0.1	-0.2 ± 0.1	2.1 ± 0.1	Amide II, 1
25	1603.0 ± 1.0	6.4 ± 1.5	-0.1 ± 0.1	0.4 ± 0.1	-0.4± 0.1	-
	1621.4 ± 1.1	10.4 ± 0.1	7.5 ± 1.8	2.5 ± 0.4	3.5 ± 0.4	C= C (Anthracene)
	1624.8 ± 0.1	9.2 ± 1.2	-1.1 ± 1.2	0.5 ± 0.7	1.9 ± 1.5	-
	1637.5 ± 3.1	14.7 ± 2.1	2.2 ± 0.2	3.2 ± 0.3	0.4 ± 0.0	Amide I, 3
	1646.9 ± 0.5	11.3 ± 0.2	1.7 ± 0.9	2.7 ± 0.6	0.5 ± 0.3	Amide I, 2
	1656.7 ± 0.1	7.7 ± 1.1	-0.6 ± 0.8	4.2 ± 1.2	0.9 ± 0.5	Amide I, 1
	1550.8 ± 0.8	14.1 ± 1.4	-0.3 ± 0.4	-0.1 ± 0.0	1.1 ± 0.5	-
	1554.2 ± 2.4	14.8 ± 0.8	2.3 ± 0.9	-0.1 ± 0.1	0.1 ± 0.0	Amide II, 2
	1562.8 ± 0.4	8.7 ± 0.8	0.1 ± 0.0	0.3 ± 0.0	0.1 ± 0.1	-
	1577.6 ± 2.1	7.5 ± 1.2	1.1 ± 0.5	0.0 ± 0.0	0.1 ± 0.2	Amide II, 3
	1598.3 ± 0.2	7.6 ± 0.3	0.3 ± 0.1	0.0 ± 0.0	0.2 ± 0.1	Amide II, 1
12	1603.0 ± 1.0	6.4 ± 1.5	0.1 ± 0.2	0.0 ± 0.0	0.1 ± 0.1	-
	1621.4 ± 1.1	10.4 ± 0.1	0.6 ± 0.1	0.2 ± 0.0	0.3 ± 0.3	C= C (Anthracene)
	1624.8 ± 0.1	9.2 ± 1.2	-0.1 ± 0.1	-0.1 ± 0.1	0.1 ± 0.1	-
	1637.5 ± 3.1	14.7 ± 2.1	0.5 ± 0.4	0.6 ± 0.2	0.2 ± 0.1	Amide I, 3
	1646.9 ± 0.5	11.3 ± 0.2	0.6 ± 0.4	1.7 ± 0.9	0.3 ± 0.2	Amide I, 2
	1656.7 ± 0.1	7.7 ± 1.1	-0.2 ± 0.1	0.4 ± 0.1	0.2 ± 0.0	Amide I, 1

Supplementary Table 3 The Global-Fitting parameters and vibrational mode assignments for the spectra of L-GAn and D-GAn monolayers in Fig. 1e. The spectra

are fitted with Lorentzian line shape function as Eq. 2. The peak position of the vibrational modes ω_q, the peak width Γ_q, and the oscillator strength factor $\chi_{\mathrm{eff},q,ijk}$ (A_{psp}) of the vibrational modes and assignment of the characteristic peaks are listed.

	Surface pressure (mN/m)	ω_q (cm^{-1})	Γ_q (cm^{-1})	A_{psp} (a.u.)	Assignment
D-GAn	40	1554.5 ± 0.5	14.6 ± 3.5	0.1 ± 0.1	Amide II, 2
		1562.4 ± 1.5	8.5 ± 0.7	2.0 ± 0.4	-
		1621.2 ± 2.1	12.2 ± 2.4	1.7 ± 0.4	C= C (Anthracene)
		1628.1 ± 3.3	7.8 ± 2.3	4.8 ± 0.6	-
		1637.4 ± 0.7	12.1 ± 1.6	1.6 ± 0.4	Amide I, 3
		1646.9 ± 0.6	17.5 ± 2.6	1.5 ± 0.1	Amide I, 2
	25	1554.5 ± 0.5	14.6 ± 3.5	0.3 ± 0.1	Amide II, 2
		1562.4 ± 1.5	8.5 ± 0.7	1.0 ± 0.3	-
		1621.2 ± 2.1	12.2 ± 2.4	0.9 ± 0.2	C= C (Anthracene)
		1628.1 ± 3.3	7.8 ± 2.3	3.7 ± 0.9	-
		1637.4 ± 0.7	12.1 ± 1.6	0.7 ± 0.2	Amide I, 3
		1646.9 ± 0.6	17.5 ± 2.6	0.6 ± 0.2	Amide I, 2
	12	1554.5 ± 0.5	14.6 ± 3.5	0.5 ± 0.2	Amide II, 2
		1562.4 ± 1.5	8.5 ± 0.7	0.6 ± 0.2	-
		1621.2 ± 2.1	12.2 ± 2.4	0.6 ± 0.2	C= C (Anthracene)
		1628.1 ± 3.3	7.8 ± 2.3	2.0 ± 0.9	-
		1637.4 ± 0.7	12.1 ± 1.6	0.4 ± 0.1	Amide I, 3
		1646.9 ± 0.6	17.5 ± 2.6	0.5 ± 0.2	Amide I, 2
L-GAn	40	1554.5 ± 0.5	14.6 ± 3.5	-0.3 ± 0.1	Amide II, 2
		1562.4 ± 1.5	8.5 ± 0.7	-1.9 ± 0.6	-
		1621.2 ± 2.1	12.2 ± 2.4	-2.0 ± 0.3	C= C (Anthracene)
		1628.1 ± 3.3	7.8 ± 2.3	-4.2 ± 1.0	-
		1637.4 ± 0.7	12.1 ± 1.6	-1.4 ± 0.3	Amide I, 3
		1646.9 ± 0.6	17.5 ± 2.6	-1.3 ± 0.2	Amide I, 2
	25	1554.5 ± 0.5	14.6 ± 3.5	0.2 ± 0.1	Amide II, 2
		1562.4 ± 1.5	8.5 ± 0.7	-1.0 ± 0.3	-
		1621.2 ± 2.1	12.2 ± 2.4	-1.1 ± 0.2	C= C (Anthracene)
		1628.1 ± 3.3	7.8 ± 2.3	-3.0 ± 0.6	-
		1637.4 ± 0.7	12.1 ± 1.6	-0.7 ± 0.2	Amide I, 3
		1646.9 ± 0.6	17.5 ± 2.6	-0.5 ± 0.2	Amide I, 2
	12	1554.5 ± 0.5	14.6 ± 3.5	-0.6 ± 0.1	Amide II, 2
		1562.4 ± 1.5	8.5 ± 0.7	-0.4 ± 0.1	-
		1621.2 ± 2.1	12.2 ± 2.4	-0.6 ± 0.2	C= C (Anthracene)
		1628.1 ± 3.3	7.8 ± 2.3	-1.7 ± 0.3	-
		1637.4 ± 0.7	12.1 ± 1.6	-0.3 ± 0.2	Amide I, 3
		1646.9 ± 0.6	17.5 ± 2.6	-0.1 ± 0.1	Amide I, 2

Supplementary Table 4 The Global-Fitting parameters and vibrational mode assignments for the spectra of L-GAn and D-GAn monolayers in Fig. 1g. The spectra are fitted with Lorentzian line shape function as Eq. 2. The peak position of the vibrational modes ω_q, the peak width Γ_q, and the oscillator strength factor $\chi_{\mathrm{eff},q,ijk}$ ($A_{s(+m)p-s(-m)p}$) of the vibrational modes and assignment of the characteristic peaks are listed.

	Surface pressure (mN/m)	ω_q (cm^{-1})	Γ_q (cm^{-1})	$A_{s\pm mp}$ (a.u.)	Assignment
L-GAn	40	1562.2 ± 1.7	8.9 ± 2.1	1.4 ± 0.1	-
		1567.4 ± 1.4	14.5 ± 0.3	1.5 ± 0.3	-
		1577.8 ± 2.3	8.7 ± 2.5	3.9 ± 1.2	Amide II, 3
		1598.5 ± 3.9	6.1 ± 1.2	-1.7 ± 0.7	Amide II, 1
		1621.0 ± 2.2	12.7 ± 2.4	-4.2 ± 1.8	C= C, (Anthracene)
		1624.7 ± 1.0	8.3 ± 2.7	5.8 ± 2.4	-
		1628.8 ± 1.7	7.0 ± 0.3	-5.2 ± 1.5	-
		1637.4 ± 2.6	12.5 ± 2.9	10.1 ± 0.8	Amide I, 3
	25	1562.2 ± 1.7	8.9 ± 2.1	-0.5 ± 0.2	-
		1567.4 ± 1.4	14.5 ± 0.3	1.2 ± 0.6	-
		1577.8 ± 2.3	8.7 ± 2.5	0.3 ± 4.5	Amide II, 3
		1598.5 ± 3.9	6.1 ± 1.2	-0.9 ± 0.7	Amide II, 1
		1621.0 ± 2.2	12.7 ± 2.4	-2.5 ± 1.2	C= C, (Anthracene)
		1624.7 ± 1.0	8.3 ± 2.7	2.6 ± 1.5	-
		1628.8 ± 1.7	7.0 ± 0.3	-4.0 ± 1.6	-
		1637.4 ± 2.6	12.5 ± 2.9	7.0 ± 1.1	Amide I, 3
	12	1562.2 ± 1.7	8.9 ± 2.1	-1.6 ± 0.2	-
		1567.4 ± 1.4	14.5 ± 0.3	1.8 ± 0.4	-
		1577.8 ± 2.3	8.7 ± 2.5	1.3 ± 0.6	Amide II, 3
		1598.5 ± 3.9	6.1 ± 1.2	-1.6 ± 1.0	Amide II, 1
		1621.0 ± 2.2	12.7 ± 2.4	-1.1 ± 0.8	C= C, (Anthracene)
		1624.7 ± 1.0	8.3 ± 2.7	2.3 ± 1.7	-
		1628.8 ± 1.7	7.0 ± 0.3	3.4 ± 1.8	-
		1637.4 ± 2.6	12.5 ± 2.9	8.9 ± 0.9	Amide I, 3

	Surface pressure (mN/m)	ω_q (cm^{-1})	Γ_q (cm^{-1})	$A_{s\pm mp}$ (a.u.)	Assignment
D-GAn	40	1562.2 ± 1.7	8.9 ± 2.1	-0.9 ± 0.1	-
		1567.4 ± 1.4	14.5 ± 0.3	-1.8 ± 0.2	-
		1577.8 ± 2.3	8.7 ± 2.5	-2.6 ± 1.2	Amide II, 3
		1598.5 ± 3.9	6.1 ± 1.2	1 ± 0.4	Amide II, 1
		1621.0 ± 2.2	12.7 ± 2.4	2.5 ± 1.0	C= C, (Anthracene)
		1624.7 ± 1.0	8.3 ± 2.7	-2.9 ± 1.1	-
		1628.8 ± 1.7	7.0 ± 0.3	6.1 ± 0.5	-
		1637.4 ± 2.6	12.5 ± 2.9	10.2 ± 3.6	Amide I, 3
	25	1562.2 ± 1.7	8.9 ± 2.1	0.3 ± 0.1	-
		1567.4 ± 1.4	14.5 ± 0.3	0.4 ± 0.2	-
		1577.8 ± 2.3	8.7 ± 2.5	-0.8 ± 1	Amide II, 3
		1598.5 ± 3.9	6.1 ± 1.2	0 ± 0.3	Amide II, 1
		1621.0 ± 2.2	12.7 ± 2.4	1.7 ± 0.5	C= C, (Anthracene)
		1624.7 ± 1.0	8.3 ± 2.7	-2.3 ± 1.8	-
		1628.8 ± 1.7	7.0 ± 0.3	4.9 ± 0.7	-
		1637.4 ± 2.6	12.5 ± 2.9	8.4 ± 2.2	Amide I, 3
	12	1562.2 ± 1.7	8.9 ± 2.1	-1 ± 0.6	-
		1567.4 ± 1.4	14.5 ± 0.3	-1.9 ± 0.3	-
		1577.8 ± 2.3	8.7 ± 2.5	-0.3 ± 0.1	Amide II, 3
		1598.5 ± 3.9	6.1 ± 1.2	1.4 ± 0.6	Amide II, 1
		1621.0 ± 2.2	12.7 ± 2.4	0.8 ± 0.5	C= C, (Anthracene)
		1624.7 ± 1.0	8.3 ± 2.7	2.3 ± 1.1	-
		1628.8 ± 1.7	7.0 ± 0.3	-4.2 ± 0.4	-
		1637.4 ± 2.6	12.5 ± 2.9	6.5 ± 1.2	Amide I, 3

Supplementary Table 5 The Global-Fitting parameters and vibrational mode assignments for the spectra of D-GAn monolayer (40 mN/m) in Supplementary Fig. 6c. The spectra are fitted with Lorentzian line shape function as Eq. 2. The peak position of the vibrational modes ω_q, the peak width Γ_q, and the oscillator strength factor $\chi_{\text{eff},q,ijk}$ $(A_{s(+m)p-s(-m)p}, A_{psp}, A_{p(+m)p-p(-m)p}, A_{spp})$ of the vibrational modes and assignment of the characteristic peaks are listed.

ω_q (cm^{-1})	Γ_q (cm^{-1})	A_{smp} (a.u.)	A_{psp} (a.u.)	A_{pmp} (a.u.)	A_{spp} (a.u.)	Assignment
1554.1 ± 1.5	14.1 ± 1.9	1.6 ± 0.5	2.8 ± 0.5	4.1 ± 0.7	6.0 ± 0.7	Amide II, 2
1562.7 ± 0.1	8.8 ± 1.2	-1.3 ± 0.1	-0.5 ± 0.2	0.0 ± 0.1	0.1 ± 0.1	-
1567.0 ± 0.6	17.3 ± 1.6	-8.4 ± 1.7	-6.3 ± 1.6	-12.5 ± 3.3	-11.5 ± 2.8	-
1577.4 ± 1.7	9.6 ± 1.8	1.3 ± 0.6	1.5 ± 0.7	6.6 ± 1.9	4.1 ± 1.4	Amide II, 3
1598.2 ± 0.7	7.0 ± 0.9	-0.8 ± 0.2	-0.1 ± 0.2	-0.1 ± 0.2	0.5 ± 0.2	Amide II, 1
1621.3 ± 2.4	11.3 ± 1.8	17.2 ± 3.5	0.5 ± 0.8	16.2 ± 3.9	-4.4 ± 1.5	C= C, (Anthracene)
1624.1 ± 1.2	10.3 ± 2.4	-14.2 ± 2.3	2.7 ± 1.5	-22.2 ± 2.9	14.5 ± 3.8	-
1628.8 ± 0.3	7.7 ± 0.5	7.6 ± 1.1	0.8 ± 0.4	5.2 ± 2.1	-9.1 ± 1.6	-
1637.2 ± 3.1	14.2 ± 3.3	-1.1 ± 1.0	1.7 ± 0.6	-0.3 ± 1.3	1.5 ± 0.7	Amide I, 3
1646.7 ± 1.0	17.2 ± 0.8	0.1 ± 0.0	1.5 ± 0.1	0.1 ± 0.1	0.0 ± 0.0	Amide I, 2

13. Global fitting of SFG spectra in C-H region

Supplementary Table 6 The Global-Fitting parameters and vibrational mode assignments for the spectra of L-GAn monolayers in different surface pressures at the C-H region. The spectra are fitted with Lorentzian line shape function as Eq. 2. The peak position of the vibrational modes ω_q, the peak width Γ_q, and the oscillator strength factor $\chi_{\mathrm{eff},q,ijk}$ (A_{ssp}, A_{sps}, A_{ppp}) of the vibrational modes and assignment of the characteristic peaks are listed. The data in Supplementary Table 6 is used to calculate the orientation angles of the terminal methyl groups of L-GAn.

Surface pressure (mN/m)	ω_q (cm^{-1})	Γ_q (cm^{-1})	A_{ssp} (a.u.)	A_{sps} (a.u.)	A_{ppp} (a.u.)	Assignment
40	2849.9 ± 0.4	12.4 ± 0.7	1.2 ± 0.1	0.0 ± 0.0	0.0 ± 0.0	CH_2-SS
	2865.2 ± 0.3	2.4 ± 0.6	0.1 ± 0.0	0.0 ± 0.0	0.0 ± 0.0	CH_2-SS
	2879.5 ± 0.1	6.8 ± 0.4	3.0 ± 0.1	-0.0± 0.0	-0.3 ± 0.0	CH_3-SS
	2921.0 ± 1.1	15.4 ± 2.3	0.0 ± 0.0	0.1 ± 0.0	0.5 ± 0.1	-
	2946.3 ± 0.2	4.4 ± 0.3	0.4 ± 0.1	0.0 ± 0.0	-0.0 ± 0.0	CH_3-FR
	2958.6 ± 0.7	4.4 ± 0.8	0.1 ± 0.0	0.2 ± 0.1	0.2 ± 0.1	-
	2968.3 ± 0.5	2.7 ± 0.1	0.0 ± 0.0	0.1 ± 0.0	1.5 ± 0.1	CH_3-AS
25	2850.6 ± 0.5	11.4 ± 0.8	1.2 ± 0.1	0.0 ± 0.0	0.0 ± 0.0	CH_2-SS
	2865.2 ± 0.3	2.4 ± 0.6	0.1 ± 0.2	0.0 ± 0.0	0.0 ± 0.0	CH_2-SS
	2879.5 ± 0.2	6.8 ± 0.4	2.1 ± 0.1	-0.0 ± 0.0	-0.1 ± 0.0	CH_3-SS
	2921.0 ± 1.1	15.4 ± 2.3	0.3 ± 0.3	0.0 ± 0.0	0.6 ± 0.0	-
	2946.3 ± 0.2	4.4 ± 0.3	0.1 ± 0.0	0.0 ± 0.0	-0.0 ± 0.1	CH_3-FR
	2958.6 ± 0.7	4.4 ± 0.8	-0.1 ± 0.2	0.1 ± 0.1	0.2 ± 0.2	-
	2968.3 ± 0.4	2.7 ± 0.1	-0.1 ± 0.1	0.0 ± 0.0	1.1 ± 0.1	CH_3-AS
12	2850.6 ± 0.5	11.4 ± 0.8	0.1 ± 0.0	0.0 ± 0.0	-0.0 ± 0.0	CH_2-SS
	2865.2 ± 0.8	2.4 ± 0.6	0.0 ± 0.0	0.0 ± 0.0	-0.0 ± 0.0	CH_2-SS
	2879.5 ± 0.1	6.8 ± 0.4	0.4 ± 0.0	-0.0 ± 0.0	0.0 ± 0.0	CH_3-SS
	2921.0 ± 1.1	15.4 ± 2.3	0.0 ± 0.0	0.0 ± 0.0	0.0 ± 0.0	-
	2946.3 ± 0.2	4.4 ± 0.3	0.0 ± 0.0	0.0 ± 0.0	0.0 ± 0.0	CH_3-FR
	2958.6 ± 0.7	4.4 ± 0.8	0.0 ± 0.0	0.0 ± 0.1	0.0 ± 0.0	-
	2968.3 ± 0.2	2.7 ± 0.1	0.0 ± 0.0	0.0 ± 0.0	0.2 ± 0.0	CH_3-AS

Supplementary Table 7 The Global-Fitting parameters and vibrational mode assignments for the spectra of D-GAn monolayers in different surface pressures at the C-H region in Fig. 2a. The spectra are fitted with Lorentzian line shape function as Eq. 2. The peak position of the vibrational modes ω_q, the peak width Γ_q, and the oscillator strength factor $\chi_{\text{eff},q,ijk}$ (A_{ssp}, A_{sps}, A_{ppp}) of the vibrational modes and assignment of the characteristic peaks are listed. The data in Supplementary Table 7 is used to calculate the orientation angles of the terminal methyl groups of D-GAn.

Surface pressure (mN/m)	ω_q (cm^{-1})	Γ_q (cm^{-1})	A_{ssp} (a.u.)	A_{sps} (a.u.)	A_{ppp} (a.u.)	Assignment
40	2850.7 ± 1.9	13.1 ± 2.7	1.3 ± 0.5	0.0 ± 0.1	-0.1 ± 0.1	CH_2-SS
	2864.2 ± 1.7	2.4 ± 2.0	0.1 ± 0.0	0.0 ± 0.1	0.0 ± 0.0	CH_2-SS
	2879.5 ± 3.3	7.4 ± 2.8	3.0 ± 0.1	-0.0 ± 0.0	-0.3 ± 0.0	CH_3-SS
	2920.4 ± 1.8	13.5 ± 2.6	0.1 ± 0.1	0.0 ± 0.0	0.3 ± 0.2	-
	2943.1 ± 1.1	3.5 ± 1.8	0.2 ± 0.1	0.0 ± 0.1	-0.0 ± 0.0	CH_3-FR
	2957.9 ± 1.6	4.5 ± 0.9	0.2 ± 0.1	0.2 ± 0.1	0.3 ± 0.1	-
	2968.3 ± 0.2	2.5 ± 0.4	-0.1 ± 0.1	-0.0 ± 0.1	1.3 ± 0.4	CH_3-AS
25	2850.1 ± 1.4	12.6 ± 0.6	1.3 ± 0.1	0.0 ± 0.1	-0.1 ± 0.0	CH_2-SS
	2865.8 ± 0.3	2.2 ± 0.7	0.1 ± 0.0	0.0 ± 0.0	0.0 ± 0.0	CH_2-SS
	2879.7 ± 1.1	7.7 ± 1.7	2.3 ± 0.3	-0.0 ± 0.1	-0.1 ± 0.0	CH_3-SS
	2920.6 ± 1.5	14.6 ± 2.8	0.3 ± 0.1	-0.0 ± 0.0	0.5 ± 0.0	-
	2945.1 ± 0.3	4.0 ± 0.5	0.1 ± 0.0	-0.0 ± 0.0	-0.0 ± 0.0	CH_3-FR
	2957.4 ± 1.1	5.4 ± 0.9	-0.1 ± 0.0	0.1 ± 0.0	0.2 ± 0.0	-
	2968.1 ± 0.1	2.5 ± 0.1	-0.0 ± 0.0	0.0 ± 0.0	1.0 ± 0.1	CH_3-AS
12	2850.1 ± 1.7	12.1 ± 1.1	0.1 ± 0.0	0.0 ± 0.0	-0.0 ± 0.0	CH_2-SS
	2864.3 ± 2.0	2.3 ± 1.0	0.0 ± 0.0	0.0 ± 0.0	0.0 ± 0.0	CH_2-SS
	2878.9 ± 1.6	7.8 ± 0.4	0.4 ± 0.1	-0.0 ± 0.1	0.0 ± 0.0	CH_3-SS
	2920.9 ± 1.5	15.2 ± 2.8	-0.0 ± 0.0	0.0 ± 0.0	0.0 ± 0.0	-
	2946.3 ± 0.6	4.5 ± 1.1	0.0 ± 0.0	0.0 ± 0.0	0.0 ± 0.0	CH_3-FR
	2959.3 ± 1.1	4.0 ± 1.0	0.0 ± 0.0	0.0 ± 0.0	0.0 ± 0.0	-
	2968.2 ± 0.4	2.5 ± 0.6	0.0 ± 0.0	0.0 ± 0.0	0.2 ± 0.1	CH_3-AS

Supplementary Table 8 The Global-Fitting parameters and vibrational mode assignments for the spectra of L-GAn and D-GAn monolayers in Fig. 2b. The spectra are fitted with Lorentzian line shape function as Eq. 2. The peak position of the vibrational modes ω_q, the peak width Γ_q, and the oscillator strength factor $\chi_{\mathrm{eff},q,ijk}$ ($A_{s(+m)p-s(-m)p}$) of the vibrational modes and assignment of the characteristic peaks are listed.

	Surface pressure (mN/m)	ω_q (cm^{-1})	Γ_q (cm^{-1})	$A_{s\pm mp}$ (a.u.)	Assignment
L-GAn	40	2849.6 ± 1.0	10.8 ± 1.4	-0.3 ± 0.0	CH_2-SS
		2865.4 ± 0.5	3.2 ± 1.0	-0.1 ± 0.0	CH_2-SS
		2878.6 ± 1.3	5.6 ± 0.9	-0.4 ± 0.3	CH_3-SS
		2944.4 ± 0.8	4.3 ± 1.1	-0.1 ± 0.0	CH_3-FR
	25	2849.6 ± 1.0	10.8 ± 1.4	-0.3 ± 0.0	CH_2-SS
		2865.4 ± 0.5	3.2 ± 1.0	-0.1 ± 0.0	CH_2-SS
		2878.6 ± 1.3	5.6 ± 0.9	-0.2 ± 0.2	CH_3-SS
		2944.4 ± 0.8	4.3 ± 1.1	-0.0 ± 0.0	CH_3-FR
	12	2849.6 ± 1.0	10.8 ± 1.4	-0.1 ± 0.0	CH_2-SS
		2865.4 ± 0.5	3.2 ± 1.0	-0.0 ± 0.0	CH_2-SS
		2878.6 ± 1.3	5.6 ± 0.9	-0.0 ± 0.0	CH_3-SS
		2944.4 ± 0.8	4.3 ± 1.1	-0.0 ± 0.0	CH_3-FR
D-GAn	40	2849.6 ± 1.0	10.8 ± 1.4	0.4 ± 0.1	CH_2-SS
		2865.4 ± 0.5	3.2 ± 1.0	0.1 ± 0.0	CH_2-SS
		2878.6 ± 1.3	5.6 ± 0.9	0.6 ± 0.3	CH_3-SS
		2944.4 ± 0.8	4.3 ± 1.1	0.1 ± 0.0	CH_3-FR
	25	2849.6 ± 1.0	10.8 ± 1.4	0.2 ± 0.0	CH_2-SS
		2865.4 ± 0.5	3.2 ± 1.0	0.1 ± 0.0	CH_2-SS
		2878.6 ± 1.3	5.6 ± 0.9	0.3 ± 0.2	CH_3-SS
		2944.4 ± 0.8	4.3 ± 1.1	0.1 ± 0.0	CH_3-FR
	12	2849.6 ± 1.0	10.8 ± 1.4	0.1 ± 0.0	CH_2-SS
		2865.4 ± 0.5	3.2 ± 1.0	0.0 ± 0.0	CH_2-SS
		2878.6 ± 1.3	5.6 ± 0.9	0.0 ± 0.0	CH_3-SS
		2944.4 ± 0.8	4.3 ± 1.1	0.0 ± 0.0	CH_3-FR

14. Synthesis of L-/D-GAn

L-GAn and D-GAn were synthesized by Liu group, and the synthesis method is as follows:[40-42]

N, N′- bis (octadecyl)- L-/D-Boc-glutamic diamide (L-/D-BG):[40] Boc-L-/D-glutamic acid (2.47 g, 0.01 mol) and octadecylamine (5.39, 0.02 mol) were mixed in a 250 mL flask and dichloromethane (200 mL) was added. Then 1-ethyl-3-(3-dimethyllaminopropyl) carbodiimide hydrochloride (EDC·HCl) (4.02 g, 0.022 mol) and 1-hydroxybenzotrizole (HOBt, 2.97 g, 0.022 mol) were added to the mixture, and the obtained mixture was stirred at room temperature for 72 hours. The obtained white solid was isolated by filtration and washed three times with dichloromethane. The crude product was dissolved in THF and precipitated by water. A fine white solid was obtained (6.5 g, 87% yield).

N, N′- bis (octadecyl)-L-/D-amino-glutamic diamide (L-/D-GAm):[41] L-/D-BG (3.575 g, 4.77 mmol) in CH_2Cl_2 (50 mL) and trifluoroacetic acid (TFA; 8 mL) was stirred at room temperature for 3 h. Then, after removal of CH_2Cl_2 and excess TFA using a rotary evaporator, the remains were dissolved in tetrahydrofuran (THF), and subsequently poured into the prepared saturated $NaHCO_3$ aqueous solution to get a white solid suspension. This was filtered and vacuum dried to give the crude product (2.990 g). Recrystallization in THF (3×80 mL) afforded the white pure product (2.600 g, 83.95 %).

N, N′-bis (octadecyl)-L-/D-(anthracene-9-carboxamide)-glutamic diamide (L-/D-GAn):[42] 1.95 g L-GAm / D-GAm (3 mmol) was dissolved in anhydrous $CHCl_3$ at 0 °C, then 2 mL anhydrous trimethylamine was added. Then 3 mmol 9-anthracene carbonyl chloride dissolved in 20 mL anhydrous $CHCl_3$ was dropped into the system. The reaction mixture was stirred at 0 °C for 12 h. After the reaction, the solvent was removed under vacuum evaporation to get a yellow crude product. The solid was then further purified by recrystallization three times in ethanol to yield light yellow powder (1.1 g, yield: 43%).

15. Supplementary References

1.Wang, J., Clarke, M. L. & Chen, Z. Polarization mapping: A method to improve sum frequency generation spectral analysis. *Analytical Chemistry* **76**, 2159-2167 (2004).

2.Lambert, A. G., Davies, P. B. & Neivandt, D. J. Implementing the theory of sum frequency generation vibrational spectroscopy: A tutorial review. *Applied Spectroscopy Reviews* **40**, 103-145 (2005).

3.Hirose, C., Yamamoto, H., Akamatsu, N. & Domen, K. Orientation Analysis by Simulation of Vibrational Sum-Frequency Generation Spectrum-CH Stretching Bands of the Methyl-Group. *Journal of Physical Chemistry* **97**, 10064-10069 (1993).

4.Dick, B., Gierulski, A., Marowsky, G. & Reider, G. A. Determination of the Nonlinear Optical Susceptibility X(2) of Surface-Layers by Sum and Difference Frequency Generation in Reflection and Transmission. *Applied Physics B-Photophysics and Laser Chemistry* **38**, 107-116 (1985).

5.Hirose, C., Akamatsu, N. & Domen, K. Formulas for the analysis of the surface SFG spectrum and transformation coefficients of cartesian SFG tensor components. *Applied Spectroscopy* **46**, 1051-1072 (1992).

6.Rocha-Mendoza, I. et al. Sum frequency vibrational spectroscopy: The molecular origins of the optical second-order nonlinearity of collagen. *Biophysical Journal* **93**, 4433-4444 (2007).

7.Leiger, K. et al. Controlling Photosynthetic Excitons by Selective Pigment Photooxidation. *The Journal of Physical Chemistry B* **123**, 29-38 (2019).

8.Moad, A. J. & Simpson, G. J. A unified treatment of selection rules and symmetry relations for sum-frequency and second harmonic spectroscopies. *Journal of Physical Chemistry B* **108**, 3548-3562 (2004).

9.Ganim, Z. et al. Amide I two-dimensional infrared Spectroscopy of proteins. *Accounts of Chemical Research* **41**, 432-441 (2008).

10.Barth, A. & Zscherp, C. What vibrations tell us about proteins. *Quarterly Reviews of Biophysics* **35**, 369-430 (2002).

11.Nguyen, K. T., Le Clair, S. V., Ye, S. & Chen, Z. Orientation Determination of Protein Helical Secondary Structures Using Linear and Nonlinear Vibrational Spectroscopy. *Journal of Physical Chemistry B* **113**, 12169-12180 (2009).

12.Chen, X., Wang, J., Boughton, A. P., Kristalyn, C. B. & Chen, Z. Multiple orientation of melittin inside a single lipid bilayer determined by combined vibrational spectroscopic studies. *Journal of the American Chemical Society* **129**, 1420-1427 (2007).

13.Fu, L. et al. Characterization of Parallel beta-Sheets at Interfaces by Chiral Sum Frequency Generation Spectroscopy. *Journal of Physical Chemistry Letters* **6**, 1310-1315 (2015).

14.Fu, L., Zhang, Y., Wei, Z.-H. & Wang, H.-F. Intrinsic Chirality and Prochirality at Air/R-(+)- and S-(-)-Limonene Interfaces: Spectral Signatures With Interference Chiral Sum-Frequency Generation Vibrational Spectroscopy. *Chirality* **26**, 509-520 (2014).

15.Miao, W., Yang, D. & Liu, M. Multiple-Stimulus-Responsive Supramolecular Gels and Regulation of Chiral Twists: The Effect of Spacer Length. *Chemistry-a European Journal* **21**, 7562-7570 (2015).

16.Zhang, Y. et al. Circularly Polarized Luminescence from a Pyrene-Cyclodextrin Supra-Dendron. *Langmuir* **34**, 5821-5830 (2018).

17.Tatulian, S. A. FTIR analysis of proteins and protein-membrane interactions. *Methods Mol. Biol. (Clifton N.J.)* **2003**, 281–325 (2019).

18.Barth, A. & Zscherp, C. What vibrations tell us about proteins. *Q. Rev. Biophys.* **35**, 369–430 (2002).

19.Tan, J., Zhang, J., Luo, Y. & Ye, S. Misfolding of a human islet amyloid polypeptide at the lipid membrane populates through beta-sheet conformers without involving alpha-helical intermediates. *J. Am.*

Chem. Soc. **141**, 1941–1948 (2019).
20.Fu, L., Xiao, D., Wang, Z., Batista, V. S. & Yan, E. C. Y. Chiral sum frequency generation for in situ probing proton exchange in antiparallel beta-sheets at interfaces. *J. Am. Chem. Soc.* **135**, 3592–3598 (2013).
21.Krimm, S. & Bandekar, J. Vibrational spectroscopy and conformation of peptides, polypeptides, and proteins. *Adv. Protein Chem.* **38**, 181–364 (1986).
22.Wang, Z. et al. A narrow amide I vibrational band observed by sum frequency generation spectroscopy reveals highly ordered structures of a biofilm protein at the air/water interface. *Chemical Communications* **52**, 2956-2959 (2016).
23.Wang, J., Chen, X. Y., Clarke, M. L. & Chen, Z. Detection of chiral sum frequency generation vibrational spectra of proteins and peptides at interfaces in situ. *Proceedings of the National Academy of Sciences of the United States of America* **102**, 4978-4983 (2005).
24.Perry, J. M., Moad, A. J., Begue, N. J., Wampler, R. D. & Simpson, G. J. Electronic and vibrational second-order nonlinear optical properties of protein secondary structural motifs. *Journal of Physical Chemistry B* **109**, 20009-20026 (2005).
25.Yan, E. C. Y., Wang, Z. & Fu, L. Proteins at Interfaces Probed by Chiral Vibrational Sum Frequency Generation Spectroscopy. *Journal of Physical Chemistry B* **119**, 2769-2785 (2015).
26.Xu, Y.-y. et al. Inhomogeneous and Spontaneous Formation of Chirality in the Langmuir Monolayer of Achiral Molecules at the Air/Water Interface Probed by In Situ Surface Second Harmonic Generation Linear Dichroism. *The Journal of Physical Chemistry C* **113**, 4088-4098 (2009).
27.Carr, J. K., Wang, L., Roy, S. & Skinner, J. L. Theoretical Sum Frequency Generation Spectroscopy of Peptides. *Journal of Physical Chemistry B* **119**, 8969-8983 (2015).
28.Krimm, S. & Bandekar, J. Vibrational Spectroscopy and Conformation of Peptides, Polypeptides, and Proteins. *Advances in Protein Chemistry* **38**, 181-364 (1986).
29.Nguyen, K. T., King, J. T. & Chen, Z. Orientation Determination of Interfacial beta-Sheet Structures in Situ. *Journal of Physical Chemistry B* **114**, 8291-8300 (2010).
30.Yang, C., Chen, P., Meng, Y. & Liu, M. Spreading Films of Anthracene-Containing Gelator Molecules at the Air/Water Interface: Nanorod and Circularly Polarized Luminescence. *Langmuir* **35**, 2772-2779 (2019).
31.Haupert, L. M. & Simpson, G. J. Chirality in Nonlinear Optics. *Annual Review of Physical Chemistry* **60**, 345-365 (2009).
32.Eisenthal, K. B. Liquid interfaces probed by second-harmonic and sum-frequency spectroscopy. *Chemical Reviews* **96**, 1343-1360 (1996).
33.Shen, Y. R. & Ostroverkhov, V. Sum-frequency vibrational spectroscopy on water interfaces: Polar orientation of water molecules at interfaces. *Chemical Reviews* **106**, 1140-1154 (2006).
34.Zhuang, X., Miranda, P. B., Kim, D. & Shen, Y. R. Mapping molecular orientation and conformation at interfaces by surface nonlinear optics. *Physical Review B* **59**, 12632-12640 (1999).
35.Wang, H. F., Gan, W., Lu, R., Rao, Y. & Wu, B. H. Quantitative spectral and orientational analysis in surface sum frequency generation vibrational spectroscopy (SFG-VS). *International Reviews in Physical Chemistry* **24**, 191-256 (2005).
36.Chen, X. Y., Wang, J., Sniadecki, J. J., Even, M. A. & Chen, Z. Probing alpha-helical and beta-sheet structures of peptides at solid/liquid interfaces with SFG. *Langmuir* **21**, 2662-2664 (2005).
37.Li, Y. et al. Ordering effects of cholesterol on sphingomyelin monolayers investigated by high-resolution broadband sum-frequency generation vibrational spectroscopy. *Chinese Chemical Letters* **29**,

357-360 (2018).
38.Polzi, L. Z., Daidone, I. & Amadei, A. A Theoretical Reappraisal of Polylysine in the Investigation of Secondary Structure Sensitivity of Infrared Spectra. *Journal of Physical Chemistry B* **116**, 3353-3360 (2012).
39.Wang, H.-F., Velarde, L., Gan, W. & Fu, L. Quantitative Sum-Frequency Generation Vibrational Spectroscopy of Molecular Surfaces and Interfaces: Lineshape, Polarization, and Orientation. *Annual Review of Physical Chemistry* **66**, 189-216 (2015).
40.Li, Y., Wang, T. & Liu, M. Gelating-induced supramolecular chirality of achiral porphyrins: chiroptical switch between achiral molecules and chiral assemblies. *Soft Matter* **3**, 1312-1317 (2007).
41.Zhu, X., Li, Y., Duan, P. & Liu, M. Self-Assembled Ultralong Chiral Nanotubes and Tuning of Their Chirality Through the Mixing of Enantiomeric Components. *Chemistry – A European Journal* **16**, 8034-8040 (2010).
42.Yang, D., Duan, P. & Liu, M. Dual Upconverted and Downconverted Circularly Polarized Luminescence in Donor–Acceptor Assemblies. *Angewandte Chemie International Edition* **57**, 9357-9361 (2018).
